# Astrocytic and neuronal accumulation of elevated extracellular K^+^ with a 2/3 K^+^/Na^+^ flux ratio—consequences for energy metabolism, osmolarity and higher brain function

**DOI:** 10.3389/fncom.2013.00114

**Published:** 2013-08-22

**Authors:** Leif Hertz, Junnan Xu, Dan Song, Enzhi Yan, Li Gu, Liang Peng

**Affiliations:** Department of Clinical Pharmacology, China Medical UniversityShenyang, China

**Keywords:** action potential, astrocyte, computational analysis, K_ir_ channel, Na^+^,K^+^-ATPase, neuron, brain potassium homeostasis, slow neuronal hyperpolarization

## Abstract

Brain excitation increases neuronal Na^+^ concentration by 2 major mechanisms: (i) Na^+^ influx caused by glutamatergic synaptic activity; and (ii) action-potential-mediated depolarization by Na^+^ influx followed by repolarizating K^+^ efflux, increasing extracellular K^+^ concentration. This review deals mainly with the latter and it concludes that clearance of extracellular K^+^ is initially mainly effectuated by Na^+^,K^+^-ATPase-mediated K^+^ uptake into astrocytes, at K^+^ concentrations above ~10 mM aided by uptake of Na^+^,K^+^ and 2 Cl^−^ by the cotransporter NKCC1. Since operation of the astrocytic Na^+^,K^+^-ATPase requires K^+^-dependent glycogenolysis for stimulation of the intracellular ATPase site, it ceases after normalization of extracellular K^+^ concentration. This allows K^+^ release via the inward rectifying K^+^ channel K_ir_4.1, perhaps after trans-astrocytic connexin- and/or pannexin-mediated K^+^ transfer, which would be a key candidate for determination by synchronization-based computational analysis and may have signaling effects. Spatially dispersed K^+^ release would have little effect on extracellular K^+^ concentration and allow K^+^ accumulation by the less powerful neuronal Na^+^,K^+^-ATPase, which is not stimulated by increases in extracellular K^+^. Since the Na^+^,K^+^-ATPase exchanges 3 Na^+^ with 2 K^+^, it creates extracellular hypertonicity and cell shrinkage. Hypertonicity stimulates NKCC1, which, aided by β-adrenergic stimulation of the Na^+^,K^+^-ATPase, causes regulatory volume increase, furosemide-inhibited undershoot in [K^+^]_e_ and perhaps facilitation of the termination of slow neuronal hyperpolarization (sAHP), with behavioral consequences. The ion transport processes involved minimize ionic disequilibria caused by the asymmetric Na^+^,K^+^-ATPase fluxes.

## Introduction

The Na^+^,K^+^-ATPase is now known to mediate most “clearance” of the elevated extracellular K^+^ concentration, resulting from neuronal excitation, in some situations aided by the Na^+^,K^+^, 2Cl^−^ and water cotransporter NKCC1. This raises a huge question, because the Na^+^,K^+^-ATPase mediates influx of 2 K^+^ in exchange for 3 Na^+^, whereas action potential propagation following Hodgkin/Huxley kinetics is based on equal Na^+^ efflux and K^+^ influx. The stoichiometry between Na^+^ influx and a possibly Ca^2+^-activated K^+^ efflux following NMDA-mediated glutamatergic stimulation is unknown and even less information is available about other types of glutamatergic stimulation. This paper will discuss the possibilities that (i) the asymmetry between Na^+^,K^+^-ATPase-mediated Na^+^ and K^+^ fluxes may be the direct cause of a known cellular shrinkage following neuronal activity; (ii) subsequent regulatory volume increase mediated by NKCC1 (known to serve such a function) is a major contributor to the undershoot following intense stimulation; and (iii) transmitter-mediated acceleration of regulatory volume increase may be of importance for effects of these transmitters on slow afterhyperpolarization (sAHP) and thus neuronal excitability. These suggestions are based on a large amount of experimental observations by others and by ourselves, which first will be reviewed in some detail.

## Neuronal excitation, metabolic increase, restoration of Na^+^/K^+^ distribution and volume regulation

The classical finding that energy demand and energy production are connected by the ability of adenosine diphosphate (ADP) to stimulate oxidative phosphorylation (Chance and Williams, 1955) provides a large part of the explanation why Na^+^,K^+^-ATPase mediated transport stimulates energy production in the brain. However, an increase in cytosolic and thus also mitochondrial Ca^2+^ concentration (Szabadkai and Duchen, 2008) can also contribute directly to the mitochondrial stimulation at least in vertebrates (Denton, 2009) at highly elevated extracellular K^+^ concentrations (see below). Ca^2+^-mediated stimulation may also play a major role, e.g., in connection with transmitter activity. Excitatory brain function depends upon many processes disturbing Na^+^ and K^+^ distribution across cell membranes, and therefore requires that these ionic “disturbances” subsequently are corrected by active transport. Excitation causes (i) Na^+^ influx into neurons in response to glutamatergic ionotropic stimulation and into astrocytes during Na^+^ gradient-driven transmitter reuptake; and (ii) action potential-mediated neuronal depolarization by Na^+^ influx. It raises extracellular K^+^ concentration through: (i) outward K^+^ flux though neuronal postsynaptic glutamate receptors and astrocytic counter-transport of K^+^ during glutamate reuptake; (ii) action potential-mediated repolarization by K^+^ efflux; and (iii) K^+^ efflux from excited GABA-ceptive neurons.

There is convincing evidence that the neuronal Na^+^ entry and subsequent K^+^ exit (Hodgkin and Huxley, 1952) during the action potential lead to sizeable increases in extracellular K^+^ concentration (Lothman et al., 1975; Dietzel et al., 1982; Ransom et al., 2000; Xiong and Stringer, 2000; D'Ambrosio et al., 2002). Extracellular K^+^ increase inhibits neuronal activity, shown by block of nerve conduction during anoxia and its reversal by replacement of K^+^-enriched extracellular medium with *anoxic* medium containing a normal K^+^ concentration (Shanes, 1951). Restoration of normal extracellular K^+^ concentration is therefore urgently needed. The restorative process may include an initial astrocytic uptake (MacAulay and Zeuthen, 2012) followed by trans-astrocytic connexin- and/or pannexin-mediated K^+^ transfer (Scemes and Spray, 2012) and K^+^ re-release (with no certain information about potentially co-travelling ionic species), and a second Na^+^,K^+^-ATPase-mediated K^+^ uptake into neurons (Bay and Butt, 2012), restoring their K^+^ content.

Especially after intense stimulation the Na^+^,K^+^-ATPase mediated events may create extracellular hypertonicity due to the Na^+^,K^+^-ATPase's 2/3 ratio between K^+^ and Na^+^ fluxes (Thomas, 1972; Clarke et al., 1989), vs. similarity between the amounts of Na^+^ leaving and those of K^+^ entering the cell during the action potential (Moujahid and d'Anjou, 2012; Moujahid pers. Inf.). In response to a resulting, possibly mainly astrocytic shrinkage, regulatory volume increase can occur by active uptake of Na^+^,K^+^, 2 Cl^−^ and water, mediated by the Na^+^,K^+^-ATPase-driven cotransporter NKCC1. This creates an additional energy demand and at least contributes to the post-excitatory undershoot and possibly to termination of the slow neuronal afterhyperpolarization (sAHP). Intracellularly the consequences of imbalance between amounts of accumulated K^+^ and exited Na^+^ may be less obvious due to the larger intra- than extracellular volume, but in the long run status quo must be maintained. Supplementation of Na^+^,K^+^-ATPase activity with operation of NKCC1 at highly elevated extracellular K^+^ concentrations (>10 mM) may partly offset the intracellular consequences on ion content caused by the asymmetry of the Na^+^,K^+^-ATPase, but only in astrocytes. In these cells the promotion of K^+^ uptake together with Na^+^ and Cl^−^ leads to accumulation specifically of K^+^ and Cl^−^, because Na^+^ is re-extruded by the Na^+^,K^+^-ATPase in exchange with K^+^ (Walz and Hinks, 1986). A different NKCC1 function, regulatory volume increase at normal extracellular K^+^ concentration may be instrumental in the regulation not only of the resulting extracellular hyperosmolarity but also of intracellular ionic disequilibrium, as will be discussed later.

Synaptic, glutamatergic activity probably stimulates energy metabolism to an even larger extent than action potential propagation (Howarth et al., 2012), although transition from low to high cortical activity is accompanied by a substantial increase in relative energy consumed by action potentials vs. synaptic potentials (DiNuzzo and Giove, 2012). The high energy requirement is mainly due to entry of Na^+^ into the neurons in large amounts, increasing dendritic Na^+^ concentrations, often drastically (Bennay et al., 2008), and necessitating subsequent Na^+^ exit. As will be discussed later, this intracellular Na^+^ increase may not affect extracellular K^+^ concentration measurably or involve astrocytes. Re-uptake of transmitter glutamate occurs mainly or exclusively in astrocytes (Danbolt, 2001) and is metabolically driven by cotransport with 2 or 3 molecules of Na^+^ entering along their electrochemical gradient, whereas 1 K^+^ exits (Rauen et al., 1992; Levy et al., 1998). It therefore leads to transient intracellular accumulation of Na^+^ also in astrocytes (Bennay et al., 2008), which may help to counteract a potential deficit in Na^+^ caused by the asymmetric Na^+^/K^+^ transport by the Na^+^,K^+^-ATPase. Finally, increases in extracellular K^+^ concentrations can occur after intense GABA-ergic stimulation of CA1 hippocampal neurons, accompanied by moderate increases in intracellular Na^+^. They are critically dependent on bicarbonate-driven accumulation of Cl^−^ in pyramidal neurons and release of K^+^ by the K^+^, Cl^−^ cotransporter KCC2 (Viitanen et al., 2010).

Astrocytic/neuronal K^+^ uptake during the clearance of increased extracellular K^+^ after action potential generation, resulting extracellular hypertonicity, and regulatory volume increase perhaps mainly in astrocytes following the clearance seem all to be essential for long-term maintenance of the “milieu interne” (Bernard, 1878) following enhanced activity. This must occur within the brain, because intracerebral K^+^, like glutamate (Hawkins et al., 1995) is virtually segregated from the systemic circulation by low blood-brain barrier permeability (Bradbury et al., 1972; Kang et al., 2013). An increased metabolic demand on astrocytes during recovery from action potentials could contribute to explain why energy expenditure calculated by Howarth et al. (2012) and largely ignoring astrocytic contributions may fall short of measured energy use, as discussed by the authors.

### In summary

Several changes in extracellular K^+^ and Na^+^ concentrations result from neuronal excitation and require correction by energy-consuming processes.

## K^+^ is actively taken up in brain cells

**Figure 1:**
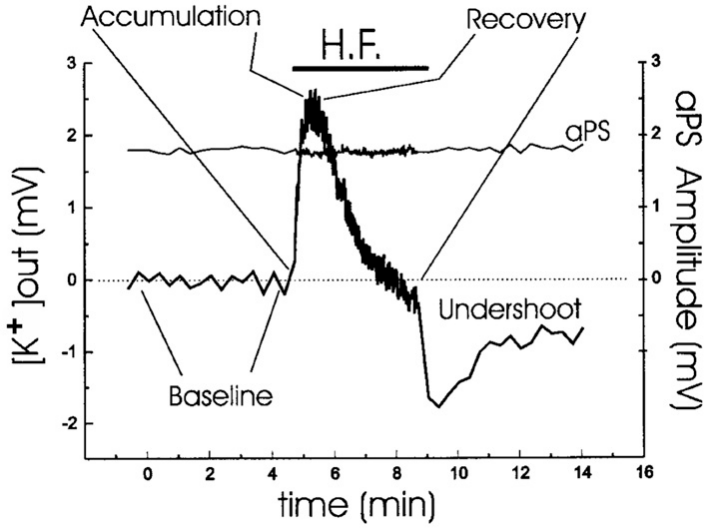
**Analysis of extracellular K^+^ homeostasis under conditions of controlled neuronal activity in hippocampal slices from young adult rats.** A stimulating electrode was placed in CA2 *stratum radiatum*. Simultaneous K^+^ activity recordings and field recordings were performed during a period of 1-Hz antidromic stimulation, indicated by the horizontal bar. The slices were normo-excitable; antidromic spike amplitude, plotted over the same time as [K^+^]_*o*_, was constant throughout the stimulating protocol; and glutamatergic activity was prevented by addition of kynurenic acid. Four phases of the extracellular K^+^-homeostasis are indicated: baseline, rapid accumulation (with a maximum K^+^ increase of ~2.5 mM), recovery during the stimulation, and subsequent undershoot reaching almost 2 mM after normalization of extracellular K^+^ concentration. From D'Ambrosio et al. (2002).

In intact brain and brain slices extracellular accumulation of K^+^ above a resting baseline occurs in response to stimulation, for example of hippocampal Schaffer collaterals at 0.05 Hz which leads to summation of the K^+^ transients evoked by each individual stimulus up to a maximal peak (Figure 1). This is followed by recovery toward baseline values, also called K^+^ clearance, and after sufficiently intense stimulation also by an undershoot in extracellular K^+^ concentration below its resting value (Lothman et al., 1975; Syková, 1992; D'Ambrosio et al., 2002). The results shown in Figure 1 were obtained during inhibition of glutamatergic activity, which accordingly did not contribute to the K^+^ dynamics.

**Figure 2:**
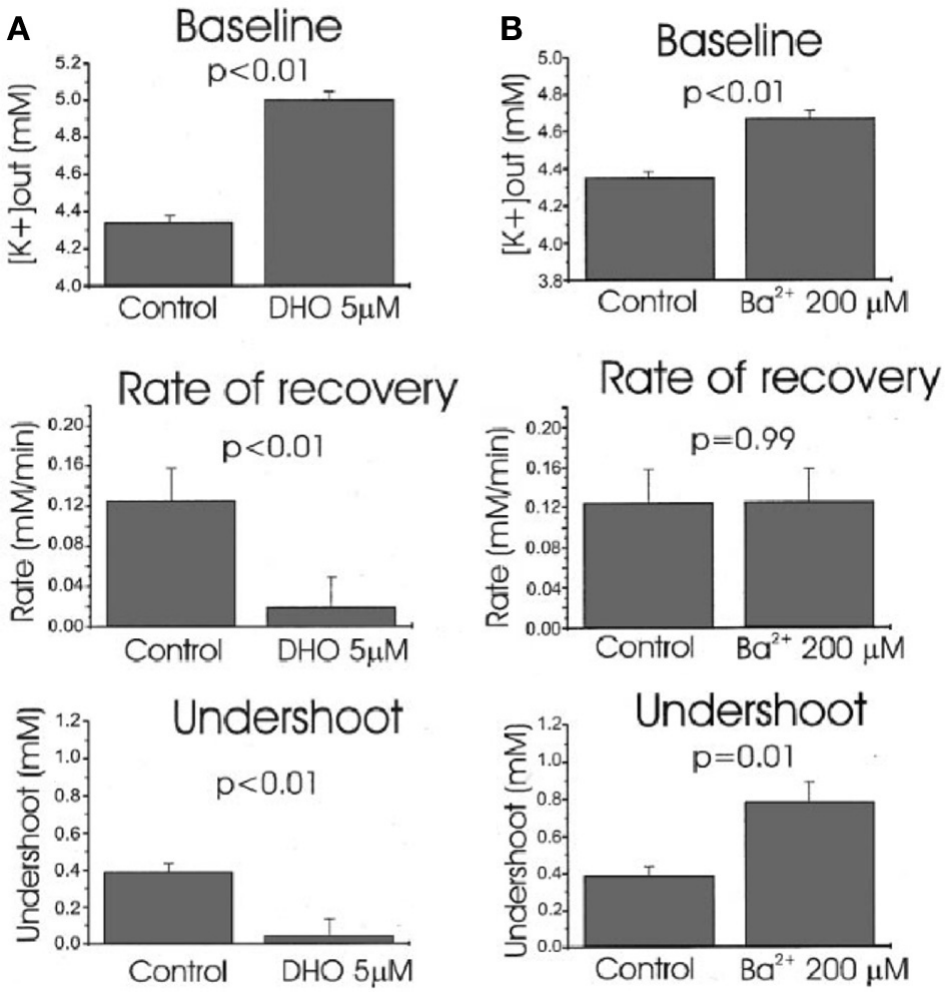
**Effects of Na^+^,K^+^-ATPase inhibition with 5 μM dihydro-ouabain, DHO (A) and K^+^ channel inhibition with 200 mM Ba^2+^**(B)** on baseline extracellular K^+^ concentration, average recovery rate during stimulation and undershoot in hippocampal brain slice experiments like that shown in Figure 1.** Both DHO and Ba^2+^ cause an increase in baseline, DHO reduces recovery rate drastically, but Ba^2+^ has no effect (*P* = 0.99 for this effect but <0.01 for all others), and the undershoot is almost abolished by DHO, but increased by Ba^2+^. The effects are consistent with profound Na^+^,K^+^-ATPase involvement in maintenance of a normal baseline, recovery after stimulation-induced K^+^ increase and undershoot, and no K^+^ channel-mediated contribution to recovery. As suggested by the authors, the Ba^2+^ effect on the undershoot suggests that outward directed K^+^ channel-mediated K^+^ transport diminishes the undershoot. The increased baseline concentration *might* suggest a less efficient spatial redistribution of K^+^. From D'Ambrosio et al. (2002).

**Figure 3:**
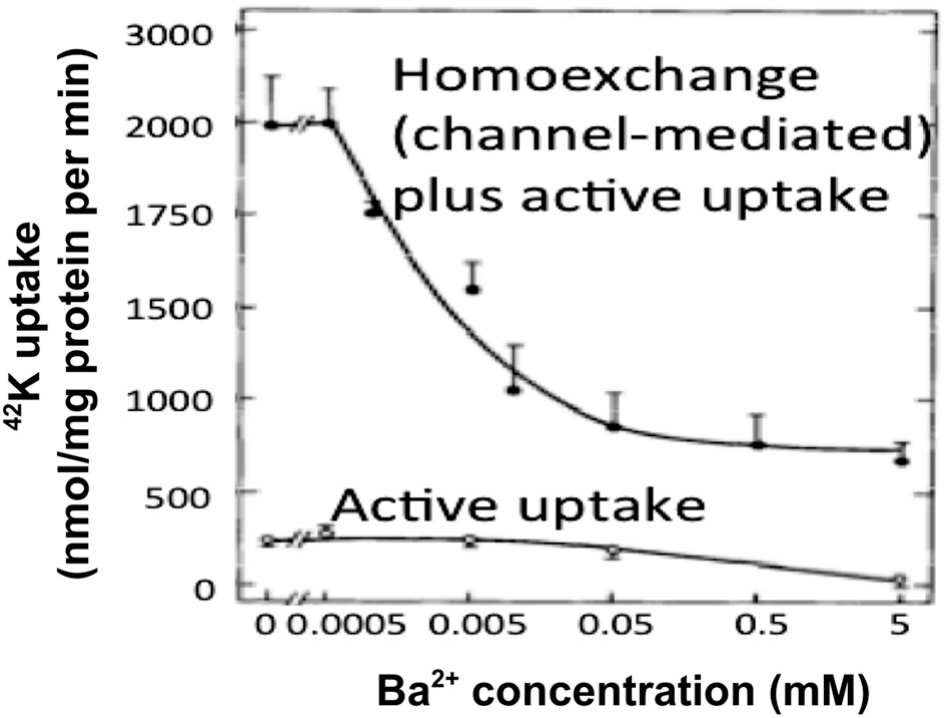
**At low extracellular concentrations Ba^2+^ specifically inhibits channel-mediated K^+^ transport as seen from the huge inhibition of ^42^K uptake into cultured astrocytes at steady state, where channel-mediated homoexchange accounts for a large fraction of the uptake.** However, at concentrations exceeding ~200 μM it also inhibits active uptake, as shown by the inhibition of the much lower uptake rate in cultures depleted for intracellular K^+^ by previous incubation in ice-cold K^+^-free medium. This is accompanied by an inhibition of Na^+^,K^+^-ATPase with similar kinetics (not shown). From Walz et al. (1984).

Ever since the first description of a spatial buffer mechanism redistributing extracellular K^+^ in the leech nervous system by Orkand et al. (1966) the presence of a similar mechanism, mediated by the astrocytic K_*ir*_4.1 channel [a constitutively active, and ATP stimulated member of the *I*nward *R*ectifier-type potassium channel family (Kubo et al., 2005)] has been postulated for K^+^ clearance in brain. This mechanism seems to be of importance in the retina (Kofuji and Newman, 2004), and this has often been extrapolated to brain. That the K^+^ clearance in mammalian brain in contrast is almost completely dependent upon active transport was shown by D'Ambrosio et al. (2002) using stimulation of Schaffer collaterals in the presence of excitatory synapse blockade to evoke purely antidromic spikes in CA3 of rat hippocampal slices without altering population spikes (Figure 1). Five micromolar di-hydro-ouabain (DHO), a Na^+^,K^+^-ATPase inhibitor, increased baseline extracellular K^+^ concentration, decreased the rate of recovery very substantially and almost abolished the undershoot, as illustrated in Figure 2A. In contrast, 200 μM Ba^2+^, a specific inhibitor of K^+^ channels at this concentration (Walz et al., 1984; Bolton et al., 2006), had no effect on the rate of recovery although it also increased baseline concentration, but it increased the undershoot (Figure 2B). Although 200 μM Ba^2+^ has little or no effect on active transport, Ba^2+^ inhibits active transport profoundly at higher concentrations (Walz et al., 1984) (Figure 3). This is accompanied by an equally potent inhibition of Na^+^,K^+^-ATPase activity, and may be the main reason for the inhibitory effect on K^+^ clearance observed by Gabriel et al. (1998) in slices from adult rats in the presence of 2 mM Ba^2+^.

**Figure 4:**
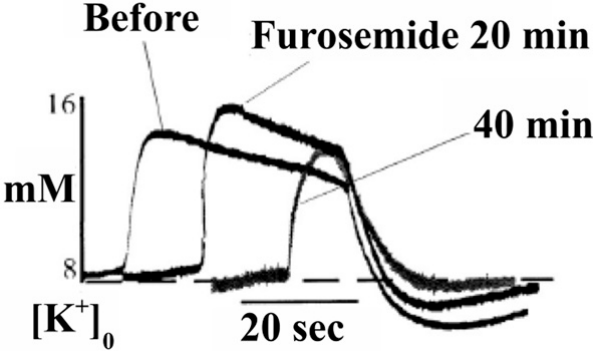
**After addition of 5 mM furosemide the already high level of evoked K^+^ concentration in these brain slice experiments, where epileptic discharges were elicited, is further increased, and field bursts are shortened.** These phenomena are related to the pathologically large elevation of the extracellular K^+^ concentration and furosemide-mediated inhibition of K^+^ clearance at these K^+^ concentration by the Na^+^,K^+^, 2Cl^−^ and water cotransporter NKCC1, and are of less relevance in the present context. However, NKCC1 is also activated by hypertonicity at normal extracellular K^+^, and may be activated after K^+^ clearance due to the asymmetry between efflux of 3 Na^+^ and influx of 2 K^+^ (see also below), thus at least contributing to the undershoot. The observation in the figure that furosemide decreases the undershoot provides support from experiments in intact tissue that astrocytes actively accumulate K^+^ during the undershoot. From Xiong and Stringer (2000).

Results essentially similar to those by D'Ambrosio et al. (2002), i.e., virtually complete dependence of K^+^ clearance on ouabain-sensitive Na^+^,K^+^-ATPase activity had previously been obtained by Xiong and Stringer (2000), using brain slices in which spontaneous epileptic activity (and higher extracellular K^+^ concentrations) were induced by incubation in a medium containing 8 mM K^+^ and no Ca^2+^. Moreover, they demonstrated that the undershoot was enhanced by furosemide (Figure 4), an inhibitor of the Na^+^,K^+^, 2Cl^−^, and water cotransporter NKCC1 (Epstein and Silva, 1985; Dawson, 1987; Hamann et al., 2010). This observation is important because NKCC1 in the adult central nervous system is absent from neuronal cell bodies, although found in dendrites (Marty et al., 2002; Deisz et al., 2011) of some neurons, whereas it is abundantly expressed in astrocytes (Kanaka et al., 2001; Mikawa et al., 2002). Astrocytes are also the cells that swell in response to high K^+^ concentrations (Zadunaisky et al., 1965; Bourke and Nelson, 1972; Møller et al., 1974). Since NKCC1 mediates secondary active transport (Pedersen et al., 2006), but needs to be driven by the transmembrane ion gradients created by the Na^+^,K^+^-ATPase, this finding is consistent with the inhibition of the undershoot by ouabain reported by D'Ambrosio et al. (2002).

### In summary

Clearance of excess extracellular K^+^ is inhibited by ouabain alkaloids, inhibiting the Na^+^,K^+^-ATPase, but not by the K_ir_ channel inhibitor Ba^2+^. The post-excitatory undershoot is inhibited by the NKCC1 inhibitor furosemide, and enhanced by Ba^2+^.

## Astrocytic contributions to extracellular K^+^ clearance

Passive glial participation in K^+^ clearance has been convincingly demonstrated in the leech (Orkand et al., 1966). In its nervous system a “spatial buffer” operating through glial cells contributes to K^+^ clearance by channel-mediated uptake of K^+^ following an electrochemical driving force into glial cells, immediate current-mediated redistribution through a glial network, and release at surroundings, which initially showed no increase (Kuffler et al., 1966). This mechanism redistributes a local increase in extracellular K^+^ concentration over a wider area, but it does not lead to any increase in intraglial K^+^ content. Although it is passive, the metabolic “cost” is a minor glial depolarization at the place of K^+^ entry. However, the concept that the initial response to excitation-induced increase in extracellular K^+^ concentration in the brain is an active astrocytic uptake (Hertz, 1965), not trans-astrocytic redistribution of K^+^, is gaining acceptance (Ransom et al., 2000; Somjen et al., 2008; Dufour et al., 2011; MacAulay and Zeuthen, 2012; Wang et al., 2012a,b). This can be seen as a further phylogenetic development of the mechanisms operating in the leech nervous system. There is also convincing evidence that channel-mediated K^+^ transport normally does not mediate K^+^ clearance in the mammalian brain cortex, where an inward directed driving force for K^+^ only exists at highly elevated extracellular concentrations (Somjen et al., 2008). At these high concentrations it can make some contribution to cellular K^+^ uptake through K_*ir*_4.1 channels. K_*ir*_4.1-mediated transport can be enhanced by aquaporin 4 (AQP4) (Padmawar et al., 2005; Soe et al., 2009). However, at the relatively low increases in extracellular K^+^ concentrations following normal physiological stimulation neither K_*ir*_4.1 (Somjen et al., 2008), nor AQP4 (Hertz et al., 2013) contribute to K^+^ clearance.

**Figure 5:**
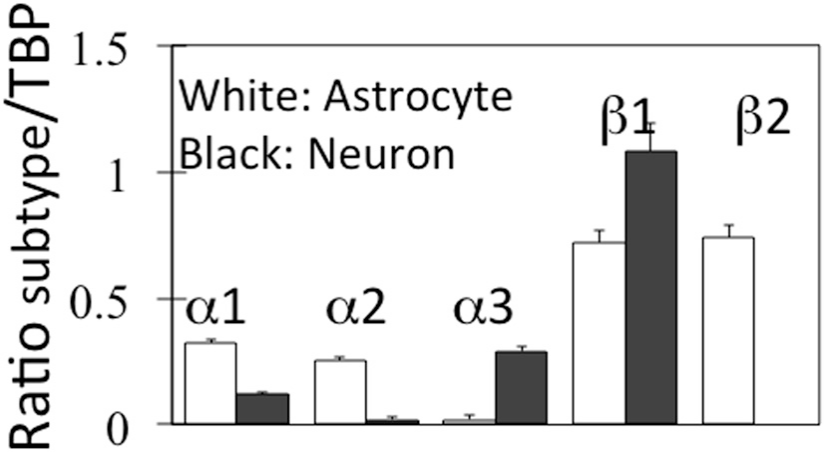
**mRNA expression of α and β subunits of the Na^+^,K^+^-ATPase in astrocytes and neurons isolated from cerebral hemispheres of normal adult mice by fluorescence-activated cell sorting (FACS).** Astrocytes were obtained from FVB/NTg(GFAP-GFP)14Mes/J transgenic mice where the astrocytes, identified by GFAP show fluorescence at one specific wavelength. Neurons were from B6.Cg-Tg(Thy1-YFPH)2Jrs/J mice, where Thy1-positive neurons fluoresce at a different wave length. The determination of mRNA was made by reverse transcription polymerase chain reaction (RT-PCR), a quantitatively more precise technique than microarray analysis. Average mRNA expression was quantitated as ratios between Na^+^,K^+^-ATPase α1, α2, α3, β1, or β2 and Tata-binding protein (TBP), used as a house-keeping gene. Moreover equal amounts of total mRNA had been applied to each gel. The methodology does not allow exact quantitative comparison between different subtypes, because of the use of different primers, but gene expression of each subtype can be quantitatively compared in astrocytes and neurons. SEM values are indicated by vertical bars. From Li et al. (2013).

Neither D'Ambrosio et al. (2002) nor Xiong and Stringer (2000) made any attempt to investigate into which cell type(s) extracellular K^+^ is accumulated. The first attempt do so in intact mammalian central nervous tissue was a study by Christopher Ransom et al. (2000) measuring activity-dependent changes in extracellular K^+^ concentration with K^+^-selective microelectrodes in the isolated optic nerve. After a 1 s, 100 Hz stimulus recovery of resting extracellular K^+^ followed a double-exponential time course with a fast time constant of 0.9 s together with a second, 5 times slower time constant (4.2 s). The rate of the fast, but not that of the slow uptake increased with increasing magnitude of the activity-dependent rise in extracellular K^+^. Both uptakes were inhibited by 50 μM strophanthidine, the slow uptake to a greater degree than the fast uptake, but they were only little affected by 200 μM Ba^2+^. Post-stimulus undershoot amplitude increased with stimulus duration, but it was independent of the peak preceding K^+^ increase. The authors suggested a model attributing the fast phase of K^+^ removal to glial Na^+^,K^+^-ATPase activity and the slower decline to K^+^ uptake via the axonal enzyme. This conclusion is in excellent agreement with characteristics of Na^+^,K^+^-ATPase activity in microdissected cerebral glial cells and synaptosomes, showing a K^+^-induced stimulation only in astrocytes (Henn et al., 1972; Grisar, 1979; Grisar et al., 1979). Moreover, gene expression of the subunits of the Na^+^,K^+^-ATPase in freshly isolated neurons and astrocytes from the mouse brain is twice as high in astrocytes for α1 and α2, whereas α3 is virtually selective for neurons and β1 is highest in neurons, which on the other hand lack β2, which is only found in astrocytes (Figure 5).

**Figure 6:**
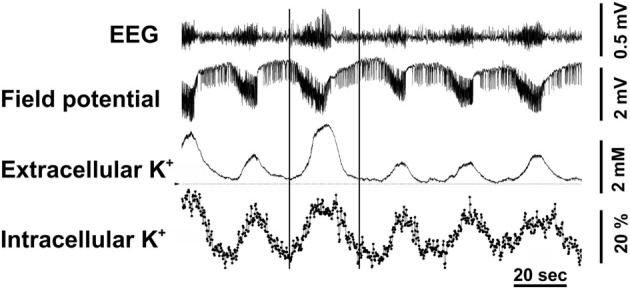
**Cortical intracellular and extracellular K^+^ variations were measured during epileptic seizures induced by topic application of penicillin on the cortex in adult cats anesthetized with ketamine–xylazine.** Extracellular K^+^ was measured by a K^+^-sensitive microelectrode and intracellular K^+^ concentration in individual cells by tapered optical fibers with a final diameter of approximately ~10 μm used to guide the excitation light deep into the tissue and to collect the fluorescence emanating from the intracellular milieu. The recorded fluorescence was that of the K^+^-sensitive indicator PBFI, applied for 2 h before starting the actual recordings. Recurrent seizures occurred every 1–1.5 min and are characterized by increased amplitude and accelerated waves in the EEG and negative direct current (DC) shift potentials in the field potentials (FP). The well-known association of such seizures with increased extracellular K^+^ concentration is shown together with virtually simultaneous increases, recorded in almost 60% of the cells and amounting to up to 60% of resting K^+^ fluorescence. The two vertical lines indicate a period during which the EEG characteristics were examined in greater detail as discussed in the text. From Dufour et al. (2011).

The conclusions by Ransom et al. (2000) are supported by elegant *in vivo* simultaneous measurements of extracellular and intracellular K^+^ concentration in the brain by Dufour et al. (2011) in the Amzica group using, respectively, microelectrodes and a fluorescent indicator of K^+^ concentration, benzfuran isophatalate (PBFI) with fluorescence intensity determination in individual cells. During seizures, associated with EEG overactivity, sustained negative direct current (DC) shifts, and increases in extracellular K^+^ concentration of 2–3 mM the authors observed phasic fluctuations of intracellular K^+^. In three fifths of the cells the discharges were associated with percentage large, but transient increases in K^+^ fluorescence (Figure 6), and in the remaining two fifths of the cells the discharges resulted in somewhat smaller decreases of fluorescence (not shown). The electroencephalographic details of the seizures, analyzed in more detail in the period between the two vertical lines in the figure were identical in the two situations. The decreases were interpreted to occur in neurons, but a neuronal increase in intracellular K^+^ concentration during seizures is virtually unthinkable, and the increases were suggested to occur in glia. The only major non-neuronal cell population in cerebral cortex is astrocytes, accounting for ~20% of the volume [reviewed by Hertz (2008)], which therefore are likely to be the cells taking up K^+^ in response to an increased extracellular K^+^ concentration.

**Figure 7:**
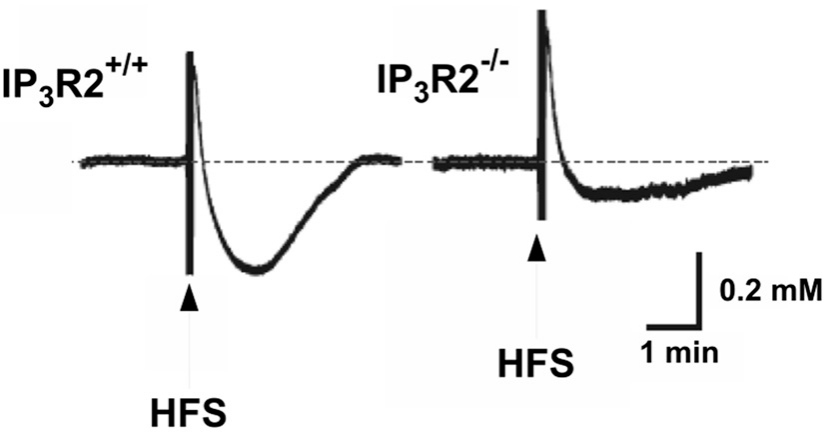
**Operation of the inositol trisphosphate receptor (IP_3_) is required both to reduce the peak K^+^ concentration after high frequency stimulation (HFS) and to evoke a sizeable undershoot after the stimulation, with the effect on the undershoot being more pronounced than that on peak concentration.** This can be seen by the larger peak K^+^ concentration in response to high frequency stimulation in rat brain slices in the IP_3_ receptor-deficient mice and the large reduction in the magnitude of the magnitude of the undershoot. The authors also demonstrated that high-frequency stimulation failed to elicit the increase in free cytosolic Ca^2+^ concentration [Ca^2+^]_i_ associated with astrocytic participation in K^+^ homeostasis in the IP_3_ receptor^−/−^ animals. From Wang et al. (2012a).

Other sophisticated *in vivo* experiments (Wang et al., 2012a,b) have shown transient decreases in brain extracellular K^+^ concentration in cerebrocortical and cerebellar slices and in cerebellum *in vivo* in response to transmitters specifically increasing free intracellular (Ca^2+^) concentration [Ca^2+^]_i_ in astrocytes. In mice with knock-out of the IP_3_ receptor, which mediates the transmitter-induced increase in [Ca^2+^]_i_, the increase in extracellular K^+^ concentration in response to high frequency stimulation was slightly increased and the undershoot almost abolished (Figure 7), reminiscent of the inhibition by furosemide shown in Figure 4. Thus, the undershoot depends critically on both NKCC1 (and Na^+^,K^+^-ATPase) activity and on IP_3_ receptor/[Ca^2+^]_i_-mediated signaling. It has already been mentioned that NKCC1 in adult brain may be less densely expressed in neurons than in astrocytes. IP_3_ receptor/[Ca^2+^]_i_-mediated signaling occurs in both neurons and astrocytes, but Wang et al. (2012a) showed that astrocyte-specific Ca^2+^ signaling (transmitter effect on a receptor normally absent in brain but introduced in transgenic animals via the GFAP promoter) could evoke a transient decrease in the extracellular K^+^ concentration. This does not mean that effects on extracellular K^+^ concentration established by astrocytic actions have no influence on neuronal activities. On the contrary, neuronal hyperpolarization secondary to astrocytic promotion of K^+^ re-accumulation led in cortical slices to suppression of baseline synaptic activity, decreased synaptic failures and increased synaptic fidelity. In intact animals the astrocyte-specific transmitter stimulation altered ECG (Wang et al., 2012a).

As discussed later in the text, the IP_3_ receptor and the increase in [Ca^2+^]_i_ by its stimulation constitute part of the astrocytic K^+^/Na^+^,K^+^-ATPase/ouabain pathway assumed to be essential for normal Na^+^,K^+^-ATPase function and thus K^+^ re-accumulation. It is also an intermediate in the β_1_-adrenergic pathway (Du et al., 2010) accelerating the operation of the astrocytic Na^+^/K^+^-ATPase/NKCC1 ion/water transport system that provides regulatory volume increase during hypertonicity, and probably contributes to the undershoot.

The very considerable rate of Na^+^ and K^+^ transport across cell membranes (Howarth et al., 2012) and the slow rate of K^+^ transport across the blood-brain barrier (Bradbury et al., 1972; Kang et al., 2013) necessitate that K^+^ initially accumulated into astrocytes must be transferred back to neurons. Bay and Butt (2012) demonstrated in rat optic nerve that the K^+^ undershoot following rapid post-stimulus clearance of elevated extracellular K^+^ recovered only slowly, especially after high stimulation frequency. Blockade of K_ir_ channels by 100 μM BaCl_2_ further slowed post-stimulus recovery of extracellular K^+^ concentration, indicating that a primary function of glial K_ir_ channels is to redistribute K^+^ to the extracellular space after its active removal. At higher levels of axonal activity, K_*ir*_ blockade also increased extracellular K^+^ accumulation, consistent with the conclusion by Somjen et al. (2008) of an inward driving force for K^+^, but only at high extracellular K^+^ concentrations.

Neurotransmitters acting on ionotropic glutamatergic (and several other ionotropic) receptors cause large Na^+^ influx into neurons leading to an increase in intracellular Na^+^ concentration (Kelly and Rose, 2010), which must be accompanied by decreases in extracellular Na^+^. Accordingly, synaptic stimulation in cerebellar or hippocampal slices causes huge tetrodotoxin-insensitive (Kuruma et al., 2003) increases in intracellular Na^+^ concentration in the spikes and dendrites of Purkinje cell (Lasser-Ross and Ross, 1992; Bennay et al., 2008) or CA1 pyramidal neurons (Rose and Konnerth, 2001). In the Purkinje cells glutamate acts mainly on AMPA receptors whereas predominantly NMDA receptors are activated in the pyramidal neurons. Peak responses in pyramidal cells are short-lasting, with time constants of about 10 s (Rose and Konnerth, 2001), but of much greater magnitude than those evoked by back-propagating action potentials (Rose et al., 1999). No evidence has been published suggesting that subsequent channel-mediated K^+^ exit should cause any increase in extracellular K^+^ concentration following the Na^+^ entry. In contrast, Broberg et al. (2007) found that increased electroencephalographic activity following systemic injection of kainate to un-anaesthetized animals has no effect on extracellular K^+^ concentration in either cortex or hippocampus until seizures occur. Possibly an intense Na^+^,K^+^-mediated Na^+^ extrusion, as demonstrated in cultured neurons (see below), might suffice to normalize intra- and extracellular Na^+^ concentration. This can be expected to increase intracellular K^+^ concentration and decrease extracellular K^+^ concentration, and a slow K^+^ release by K^+^ currents (Zorumski et al., 1989; Shah and Haylett, 2002) may become rapidly normalized.

Graded increases in extracellular K^+^ in stratum pyramidale and stratum radiatum of 2–12 mM have, however, been reported by Obrocea and Morris (2000) after 20 s of 1–10 Hz stimulation of Schaffer collaterals in hippocampal slices. They were attributable to GABA_A_ receptor activation and were interpreted as at least partly arising from co-transport with Cl^−^/HCO^−^_3_ (Autere et al., 1999). Later studies have confirmed that K^+^ responses in CA1 hippocampal neurons induced by high-frequency stimulation or GABA_A_ agonist application depend upon efflux through the K^+^, Cl^−^ cotransporter KCC2 following bicarbonate-driven accumulation of Cl^−^ in pyramidal neurons (Viitanen et al., 2010). No evidence was found of contribution by channel-mediated K^+^ efflux, and NMDA, and AMPA antagonists had only little effect on the K^+^ increase, whereas it was almost abolished by the GABA antagonist picrotoxin (Kaila et al., 1997).

### In summary

Christopher Ransom showed that clearance of the increase in extracellular K^+^ in the stimulated optic nerve occurred by two processes with different kinetic constants and correlated the most potent uptake with glial uptake and the less potent uptake with axonal uptake. Experiments by the Amzica group demonstrated an increase in intracellular K^+^ in cells, tentatively identified as glial concomitant with the increase in extracellular K^+^ during brain stimulation *in vivo*.

## Consistent and supplementary observations in cultured cells

Important results in this paper have been obtained using cultured mouse astrocytes grown using a technique that has worked well for 35 years (Hertz et al., 1978) and provided many results subsequently confirmed in the brain *in vivo* (Lange et al., 2012). Moreover, the presently reported results are consistent with literature data for K^+^ homeostasis (Ransom et al., 2000; Xiong and Stringer, 2000; D'Ambrosio et al., 2002; Somjen et al., 2008; Dufour et al., 2011) and for neuronal hyperpolarization (McCormick and Prince, 1988; Huang and Somjen, 1995, 1997; Yuan et al., 2010). However, astrocyte cultures have recently been severely criticized (Kimelberg, 2010; Foo et al., 2011). Although not recognized by these authors, protocols for culture production vary widely, and our methodology is not similar to those discussed by Foo et al. (2011), which displayed differences in mRNA expression of a multitude of genes between freshly isolated astrocytes and the cultures studied (Cahoy et al., 2008). Nor are our cultures similar to those used previously by Harold Kimelberg (Hertz et al., 1985). Recently we have compared expression of genes of interest in specific studies in freshly isolated astrocytes and these cultured astrocytes and consistently shown similar expression of several different genes for acid extruders, other transporters and glutamate receptor subtypes (Fu et al., 2012; Li et al., 2012a,b; Song et al., 2012). The gene expression of Na^+^,K^+^ATPase subtypes in freshly isolated cells which was shown in Figure 5 is also identical to that previously shown in these cultures (Peng et al., 1997, Under revision). The freshly isolated cell fractions are well suited for studies of gene expression but probably not capable of normal ion transport due to potential membrane effects caused by some of the isolation procedures.

**Figure 8:**
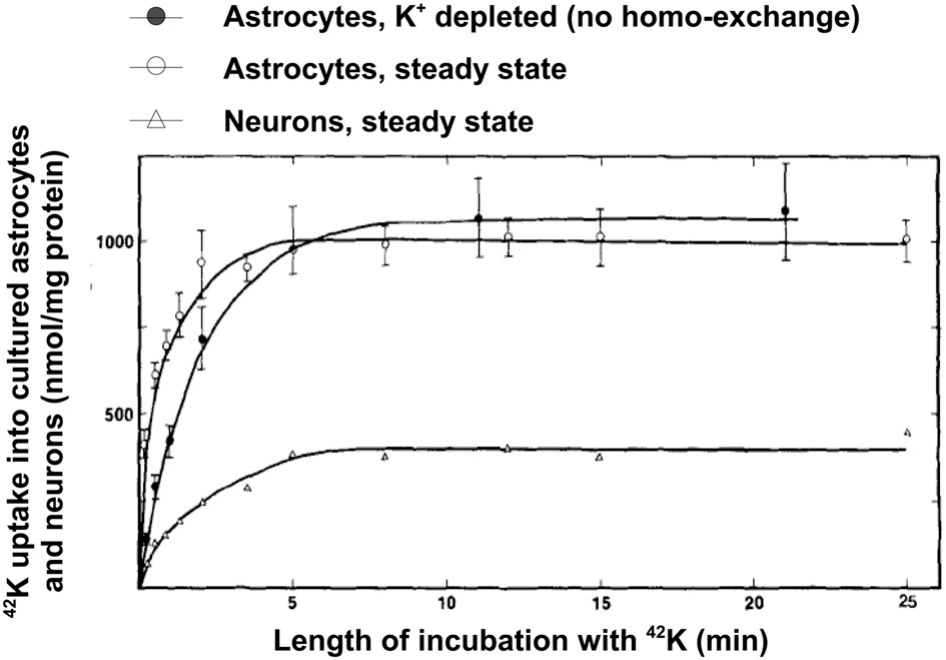
**Uptake of ^42^K in cultured cerebral astrocytes and neurons (the GABAergic cortical interneurons) at 37°C as a function of time of exposure to the isotope during incubation in tissue culture medium with 5.4 mM K^+^ (same concentration as during the culturing).** Uptake into the astrocytes was measured both at steady state (i.e., cells obtained directly from the incubator and only exposed to medium change and radio-isotope addition), where it includes channel-mediated homoexchange, and in cells depleted for intracellular K^+^ during previous incubation in ice-cold K^+^-free medium, where all uptake above an intracellular concentration of 5.4 mM must be active. Uptake into neurons was only measured at steady state, since their K^+^ permeability at a K^+^ concentration of 5.4 mM is negligible. From Hertz (1986).

A high rate of net uptake of ^42^K into primary cultures of astrocytes, which by previous incubation in ice-cold K^+^-free medium had lost virtually all intracellular K^+^ (preventing homoexchange between intra- and extracellular K^+^) was shown by Hertz (1979). As illustrated in Figure 8, the net uptake rate in these astrocytes is somewhat slower than in non-pre-incubated cells, where a channel-mediated K^+^/K^+^ homoexchange further increases uptake rate, but it is several times faster than in cultures of the GABA-ergic cerebral cortical interneurons. This difference is consistent with the differences in time constants between the two uptake components distinguished in optic nerve by Ransom et al. (2000). The plateau reached is also higher in astrocytes than in neurons indicating a higher concentration of exchangeable K^+^ (Figure 8). However, even the K^+^ uptake rate in the neurons is substantial. Assuming 100 mg protein per g tissue wet weight (probably a minimum value), an uptake of more than 100 nmol/mg protein after 1 min corresponds to a K^+^ uptake of >10 mM/per min. With a 3/2 ratio between Na^+^ and K^+^ fluxes this corresponds to a Na^+^ efflux of >15 mM/per min, which can be expected to be further increased when the intracellular Na^+^ concentration becomes elevated.

Ouabain inhibits K^+^ uptake into cultured astrocytes and neurons with relatively similar sensitivity and maximum inhibition requiring at most 1.0 mM at resting extracellular K^+^ concentration (Walz and Hertz, 1982), although ouabain sensitivity decreases with increasing K^+^ concentration, as in other tissues. Like total uptake, the ouabain-sensitive uptake was several times less intense in neurons than in astrocytes, where it reached its maximum value at an external K^+^ concentration of ~12 mM. Subtraction of the ouabain-sensitive uptake from the total uptake revealed a considerable ouabain-resistant uptake, which in astrocytes rose with increasing K^+^ concentration and substantially exceeded the maximum amount that can be accumulated by diffusion. Most of this uptake is inhibited by furosemide, indicating that it is mediated by NKCC1 (Walz and Hertz, 1984). Furosemide exerted no inhibition at low K^+^ concentrations (up to ~10–15 mM) but caused increasing inhibition at higher external K^+^ levels, reaching almost one half of the total influx at 72 mM. At extracellular K^+^ concentrations of at least 10–12 mM, Na^+^ accumulated by NKCC1 is extruded again by the Na^+^,K^+^-ATPase in a Na^+^ cycle (Walz and Hinks, 1986). This is not the case at smaller increases in extracellular K^+^ concentration, where supply of intracellular Na^+^ required for co-stimulation of the ATPase's intracellular Na^+^-sensitive site requires signaling (Xu et al., 2013). NKCC1 can also be stimulated at non-elevated K^+^ concentrations by hyperosmotic shrinkage (Akar et al., 1999; Qusous et al., 2011), where it contributes to regulatory volume increase (Hoffmann et al., 2009). In this situation there is no Na^+^ cycling, as will be demonstrated later. Thus the fate of intracellular Na^+^ accumulated into astrocytes by NKCC1 depends on the manner in which NKCC1 stimulation has occurred. However, since Na^+^,K^+^-ATPase activity is needed to create the ionic gradients driving NKCC1, and isoproterenol stimulates the astrocytic Na^+^,K^+^-ATPase, this β-adrenergic agonist can also be expected to enhance volume increase.

**Figure 9:**
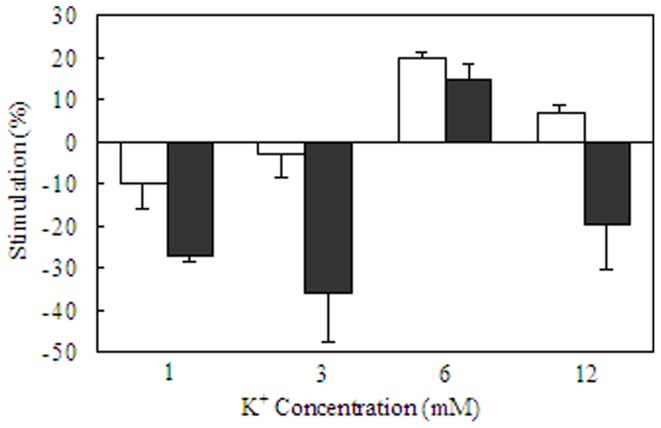
**Acute effects of elevated extracellular K^+^ concentrations and/or noradrenaline in homogenates of primary cultures of mouse astrocytes from the cerebral cortex and of primary cultures of mouse cerebral cortical neurons.** After rinsing in TRIS/HCl buffer (pH 7.5) the tissue was sonicated in similar buffer. An aliquot of the suspension was used to measure total ATPase activity in a Tris/HCL buffered saline with 5 mM MgCl_2_, 100 mM NaCl and 5–6 mM KCl. Another aliquot was added to a reaction mixture containing 172 mM Tris, 5 mM MgCl, 14 mM NaCl, and 1 mM ouabain for measurement of the activity of Mg^2+^ activated ATPase. After preincubation for 10 min at 37°C, vanadate-free adenosine triphosphate was added to a final concentration of 3 mM. The reaction was terminated by addition of trichloroacetic acid. Free phosphate was measured, using an ammonium molybdate method. ATPase activities were expressed as rates of formation of inorganic phosphate/mg protein in the homogenate per hour, and Na^+^,K^+^-ATPase activity was calculated as the difference between total ATPase activity and Mg^2+^-activated ATPase activity. Kinetics for K^+^ stimulation in each of the two cell types is shown in Table 1. The present figure shows stimulation (indicated in the upwards direction) and inhibition (indicated in the downwards direction) by 10 μM noradrenaline at K^+^ concentrations of 1, 3, 6, or 12 mM in astrocytes (white columns) and neurons (black columns). The activity in the same homogenates in the absence of any transmitter is assigned the value of 0%. Note the restriction of the noradrenaline effect to a normal K^+^ concentration and the profound inhibition of neuronal uptake at aberrant K^+^ concentrations. Results are averages ± SEM for 4–7 different homogenates. From Hajek et al. (1996).

Determination of Na^+^,K^+^-ATPase activity in homogenates from cultured mouse neurons and astrocytes (Hajek et al., 1996) has shown a higher V_max_ in the astrocytic than in the neuronal homogenate, and the affinity for K^+^ was lower in the astrocytes (higher K_m_). This is documented in Table 1, where a comparison also is made with corresponding results from astrocytic and neuronal-perikaryal cell fractions from the rabbit brain obtained using gradient centrifugation by Grisar (1979), with the latter underlined. Although all values are higher in the Grisar study [the K_m_ values perhaps unreasonably high compared with whole brain homogenates (Mercado and Hernandez, 1992)] both groups (Grisar, 1979; Grisar et al., 1979; Hajek et al., 1996) agree that the astrocytic enzyme is stimulated by increases in extracellular K^+^ concentration above its normal level. These observations are, again, consistent with the results by Ransom et al. (2000), who in addition to a faster uptake in the presumably glial component described its selective rate increase with increasing magnitude of the activity-dependent rise in extracellular K^+^. Such a difference in the sensitivity of the Na^+^,K^+^-ATPase to K^+^ (and also to Na^+^) in different cell types is made possible by different α and β subtype composition and expression of the auxiliary protein FXYD 7 (Crambert et al., 2000; Li et al., 2013) as shown in Figure 5. In both cell homogenates Na^+^,K^+^-ATPase activity could also be stimulated by noradrenaline, but the subtype receptors involved varied, being β-adrenergic in astrocytes and α_2_-adrenergic in neurons (Hajek et al., 1996; Hajek, Subbarao and Hertz, additional experiments). However, the stimulation by noradrenaline could only be observed at normal K^+^ concentrations, possibly with a small remaining effect on the astrocytic enzyme at slightly elevated K^+^ concentrations (Figure 9). In contrast, especially the neuronal Na^+^,K^+^-ATPase was markedly inhibited by noradrenaline at K^+^ concentrations below normal. A similar lack of additive effect between transmitters and elevated K^+^ concentrations has been observed for K^+^ uptake in cultured astrocytes (Wang et al., 2012a). Also, in *in vitro* intact thalamus preparations isoproterenol application causes neuronal depolarization and reduction of the slow neuronal afterhyperpolarization (sAHP) only at normal ambient K^+^ concentration, but not at elevated K^+^ concentrations (McCormick and Prince, 1988).

**Table 1:** **Kinetics (K_m_ and V_max_ values) for effects of K^+^ on Na^+^,K^+^-ATPase in astrocytes and neurons**.

**Cell type**	**Author**	**K**_**m**_ **(mM)**	**V**_**max**_ **(μmol/hr per) mg protein)**
Astrocytes	Hajek et al.	1.9 C.L.: 1.27–2.93	5.4 C.L.: 3.3–8.1
Astrocytes	Grisar	5.6 ± 2.3	13.0 ± 2.3
Neurons	Hajek et al.	0.43 ± 0.08	1.77 ± 0.06
Neurons	Grisar	2.2 ± 1.0	5.6 ± 2.1

C.L.: confidence limits. The values from Hajek et al. (1996) were obtained using cultured mouse cells and the methodology described in the legend of Figure 9. The underlined values are from Grisar (1979) using astrocytes and neuronal perikarya obtained by gradient centrifugation from the rabbit brain. Absolute values vary between the two studies, but the relation between effects on astrocytes and neurons is similar.

**Figure 10:**
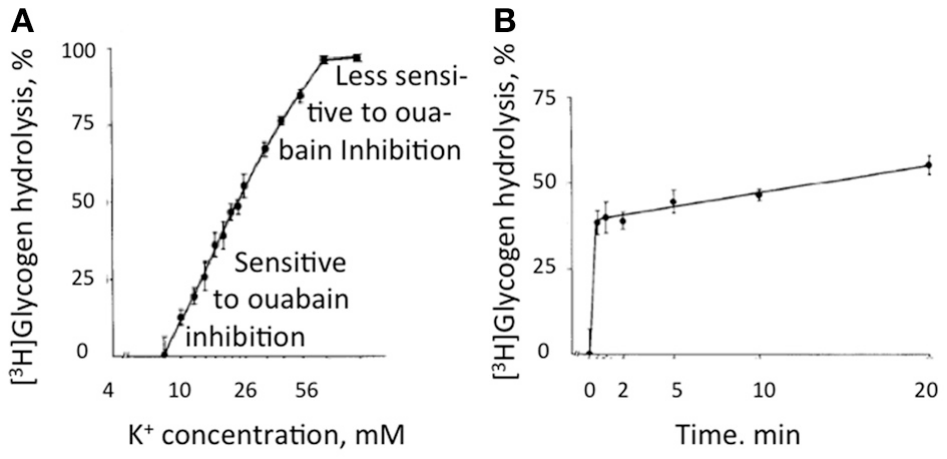
**Glycogenolysis in adult mouse brain slices incubated in a physiological saline medium with a K^+^ concentration of 3 mM, i.e., the same concentration as in the brain *in vivo*, excess of glucose, and exposure to [^3^H] glucose beginning 30 min before addition of KCl.** KCl was added to different final concentrations during the last 10 min in **(A)** and the last 20 min in **(B)** without changing medium composition. Glycogen was isolated after the experiment and remaining radioactivity measured in order to determine percentage glycogenolysis. **(A)** Glycogenolysis as a function of the extracellular K^+^ concentration. **(B)** Glycogenolysis as a function of time during exposure to an extracellular K^+^ concentration of 10 mM. At low K^+^ concentrations glycogenolysis was sensitive to ouabain inhibition, but this sensitivity was less expressed at higher K^+^ concentrations. From Hof et al. (1988).

Recently DiNuzzo et al. (2012) suggested that glycogenolysis may be essential for K^+^ uptake in astrocytes. In the brain glycogen is present and glycogenolysis occurs in astrocytes but not in neurons (Ibrahim, 1975; Richter et al., 1996). In brain slices glycogenolysis is stimulated (Figure 10) by even small increases in extracellular K^+^ (Hof et al., 1988). Elevated extracellular K^+^ concentrations also stimulate glycogenolysis in cultured astrocytes (Subbarao and Hertz, 1990), although the effect is contingent upon the culturing conditions. Thus elevated K^+^ stimulates glycogenolysis in our mature cultured astrocytes, but not in cells that have not undergone full maturation by treatment with dibutyryl cyclic AMP (Hertz and Code, 1993), a treatment presumably replacing lack of noradrenergic innervation in the developing cultures, since noradrenergic fibers from locus coeruleus mainly arrive in brain cortex after birth (Foote et al., 1983), the time when the cells were harvested. Glycogenolysis is also stimulated by several transmitters, including noradrenaline, in brain tissue (Magistretti, 1988) and in cultured astrocytes, where the main effect is exerted on β-adrenergic receptors (Subbarao and Hertz, 1990). In brain the stimulation at small increases of extracellular K^+^ concentration is inhibited by ouabain at its normal inhibitory concentrations (Hof et al., 1988).

**Figure 11:**
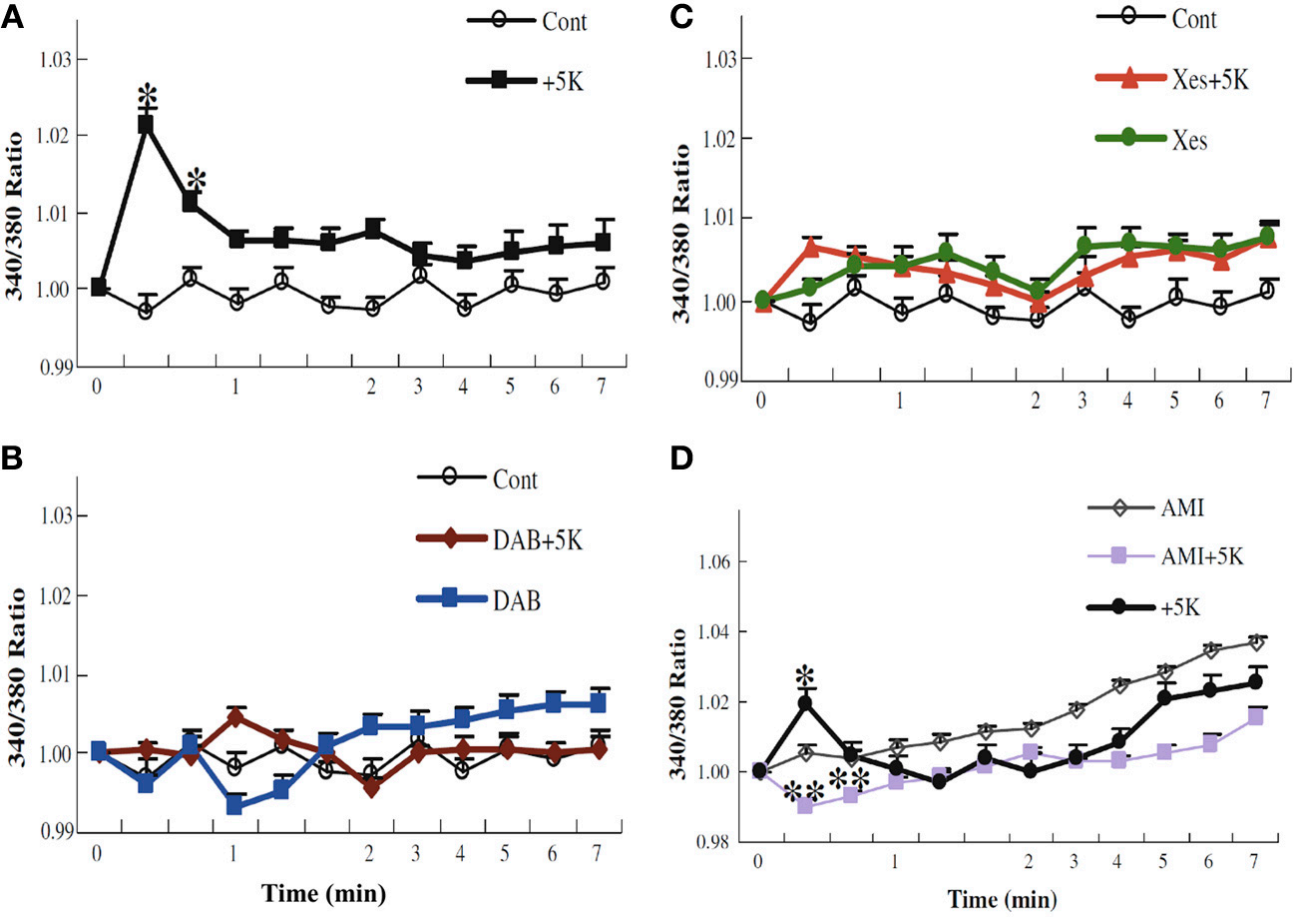
**K^+^ uptake into astrocytes measured as increase in intracellular K^+^ concentration, determined in arbitrary units based on enhanced fluorescence of the K^+^ indicator benzfuran isophthalate (PBFI) in the presence of Pluronic acid.** After incubation of PBFI-AM-loaded cells in saline solution for 2 min and subsequent wash, the cells were from zero time incubated either in similar solution or in a solution to which an additional 5 mM KCl had been added isosmotically. In some experiments, 10 mM DAB **(B)**, an inhibitor of glycogenolysis; 500 μM xestospongine **(C)**, an inhibitor of IP_3_ receptors; or 200 μM amiloride **(D)**, an inhibitor of Na^+^ channels, had been added at the time the measurement of fluorescence ratios began (2 min before K^+^ was added to some cultures). It can be seen that normally the intracellular K^+^ concentration increases after addition of 5 mM K^+^ to the incubation medium **(A)**, that this response requires glycogenolysis **(B)** and IP_3_ receptor function **(C)**, and that the K^+^ response is absent in the presence of amiloride **(D)**. Results for each condition were based on measurements of fluorescence in 40–77 individual cells from two to five separate coverslips. SEM values are indicated by vertical bars. ^*^Statistically significant (*p* < 0.05) difference from other groups at the same time period. ^**^Statistically significant (*p* < 0.05) difference from control group at the same time period. From Xu et al. (2013).

**Figure 12:**
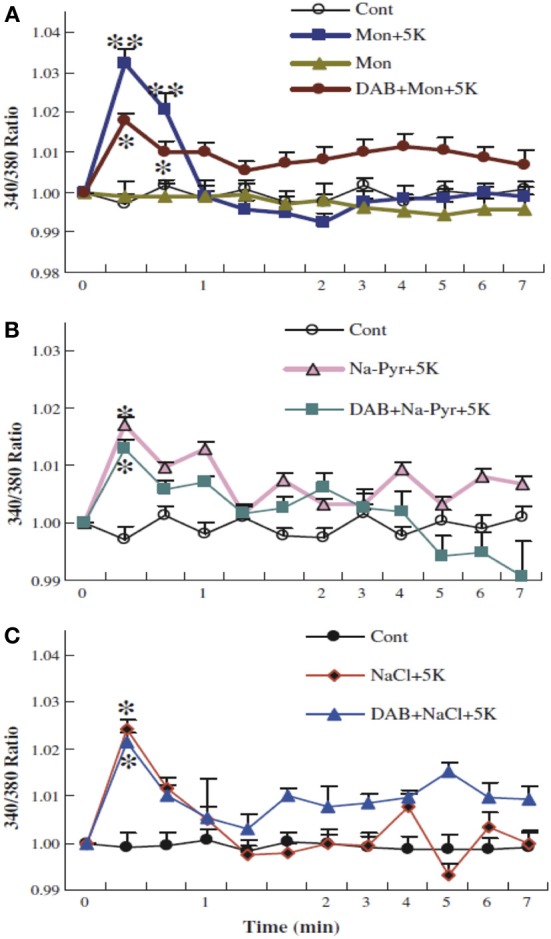
**Glycogenolysis is not required for K^+^ uptake into cultured astrocytes after addition of 5 mM K^+^, when intracellular Na^+^ has been increased.** After incubation of PBFI-AM-loaded cells in saline solution for 2 min and subsequent wash, the cells were from zero time incubated either in similar solution or in a solution to which an additional 5 mM KCl had been added as described above, with or without 10 mM DAB, and/or 0.5 μM monensin, a Na^+^/H^+^ exchanger which had been added at the time the measurement of fluorescence ratios began **(A)**. In other experiments 10 mM Na-pyruvate **(B)** or 10 mM NaCl **(C)** was added at this time, without osmotic compensation, i.e., increasing the extracellular Na^+^ concentration by 10 mM, again with or without DAB. In the presence of monensin, added Na-pyruvate or added NaCl, DAB was no longer able to inhibit K^+^ uptake, suggesting that glycogenolysis in the experiments illustrated in Figure 11 was required in order to supply Na^+^ for stimulation of the intracellular site of the Na^+^,K^+^-ATPase. Results for each condition were based on measurements of fluorescence in 29–77 individual cells from two-three separate coverslips. SEM values are indicated by vertical bars. ^*^Statistically significant (*p* < 0.05) difference from other groups at the same time period. ^**^Statistically significant (*p* < 0.05) difference from DAB + monensin + 5 K^+^ group at the same time period. From Xu et al. (2013).

Whether glycogenolysis is required for K^+^ uptake into astrocytes was tested by determining intracellular K^+^ concentration, measured in arbitrary units, in cultured astrocytes by aid of the fluorescent K^+^ sensor benzfuran isophthalate (PBFI), when the extracellular K^+^ concentration was increased by 5 mM from its resting level of 5 mM (traditionally used during the culturing, which historically began before the low extracellular K^+^ concentration in brain was known). Addition of 5 mM K^+^ was chosen, since Na^+^,K^+^-ATPase activity alone suffices for clearance of extracellular K^+^ concentrations up to 10 mM, consistent with the previously mentioned lack of furosemide on K^+^ uptake at these concentrations (Walz and Hertz, 1984). Figure 11A shows that addition of 5 mM KCl (with osmotic compensation) creates a transient highly significant peak in fluorescence, followed by a smaller, longer lasting increase. Why the peak is transient in spite of a maintained elevated extracellular K^+^ concentration is unknown. This increase is abolished by 1,4-dideoxy-1,4-imino-d-arabinitol (DAB), an inhibitor of glycogenolysis (Figure 11B), by xestospongin, an IP_3_ receptor inhibitor (Figure 11C), and by amiloride (Figure 11D), a relatively non-specific inhibitor of Na^+^ channels (Xu et al., 2013). However, DAB inhibition can be counteracted by the Na^+^/H^+^ exchanger monensin, known to increase intracellular Na^+^, or increase of the extracellular Na^+^ concentration by addition of 10 mM Na^+^-pyruvate or NaCl to the medium (Figures 12A–C). Such an increase in extracellular Na^+^ concentration enhances channel-mediated uptake of Na^+^ (Noda, 2007). Glutamate uptake in astrocytes occurs also together with uptake of Na^+^, and in the brain *in vivo* glutamatergic synaptic transmission leads to an increase in intracellular Na^+^ concentration in astrocytes of several mM (Bennay et al., 2008). However, the response is shortlasting (half-life 12 s), so that it may not be able to sustain continued astrocytic K^+^ uptake. It may also occur in astrocytes too distant from those accumulating K^+^ after action potential propagation, although Na^+^ is known to travel widely through astrocytic gap junctions (Langer et al., 2012).

Together Figures 11, 12 show that K^+^-stimulated glycogenolysis is required for Na^+^,K^+^-ATPase-mediated increase in intracellular K^+^ concentration and that this inhibition can be counteracted by stimulation of Na^+^ uptake into the cells. Further studies suggested that K^+^ binding to the Na^+^,K^+^-ATPase stimulated a pathway triggered by endogenous ouabains, operating via the IP_3_ receptor (and many other intracellular messengers) and increase in [Ca^2+^]_i_ and leading to the glycogenolysis-dependent opening of the Na^+^ channel Na_x_ (Xu et al., 2013). The effect of these endogenous ouabains can be mimicked by ordinary ouabain at nM concentrations but are abolished by higher concentrations. This abolishment may explain the ouabain-induced inhibition of glycogenolysis observed in response to small increases in extracellular K^+^ observed by Hof et al. (1988). Without opening of the Na_x_ channel (or increase of intracellular Na^+^ concentration by other means) K^+^-induced stimulation of the astrocytic Na^+^,K^+^-ATPase cannot operate, because of failing co-stimulation of the intracellular Na^+^-sensitive site of the Na^+^,K^+^-ATPase. No similar problem exists in neurons because of Na^+^ entry during excitation, and Na^+^ channel activity is not needed in astrocytes at rest (Rose et al., 1997).

Accordingly, the powerful astrocytic Na^+^,K^+^-ATPase is likely to be responsible for most K^+^ clearance as long as the extracellular K^+^ concentration is elevated, but once a resting extracellular K^+^ concentration is re-established, it becomes inactivated. This allows slower and perhaps spatially dispersed K_ir_4.1-mediated K^+^ release to occur from astrocytes without any resulting increase in extracellular K^+^ concentration and to be followed by re-uptake into neurons by the less powerful and Na^+^-regulated neuronal Na^+^,K^+^-ATPase. It is likely that this release occurs after trans-astrocytic K^+^ transport, providing signaling opportunities (Scemes and Spray, 2012) and minimizing any local increase in extracellular K^+^ concentration following Kir4.1-mediated release. This sequence of events needs further confirmation *in vivo* or in intact tissues, although both contribution by K_ir_ channels (Bay and Butt, 2012) to K^+^ homeostasis and astrocytic K^+^ uptake (Wang et al., 2012a,b) were demonstrated in intact tissue and partly *in vivo*. Moreover, the increasing acceptance that astrocytic K^+^ uptake is important for K^+^ homeostasis *in vivo* (MacAulay and Zeuthen, 2012), would not be possible without some mechanism for subsequent transfer of K^+^ to neurons.

Increases in extracellular K^+^ concentration above 10 mM could, a priori, be expected to be resistant to glycolytic inhibition on account of the proposed Na^+^ cycle between the two transporters operating at these concentrations (Walz and Hinks, 1986), but direct experiments showed this not to be the case (Xu et al., 2013). This is because activation of NKCC1 by K^+^ concentrations above 10 mM is dependent upon a different glycogenolysis-dependent pathway (depolarization-mediated opening of L-channels and consequent Ca^2+^ entry [Yan et al., 2013]). The increased [Ca^2+^]_i_ in turn increases intramitochondrial Ca^2+^ concentration (Szabadkai and Duchen, 2008), which can exert a stimulation of energy metabolism by direct activation of mitochondrial enzymes (Griffiths and Rutter, 2009). This mechanism is generally thought to be relatively little involved during normal activity. This may not necessarily be so. Since Na^+^ accumulated via NKCC1 at the highly elevated extracellular K^+^ concentrations is re-extruded by the Na^+^,K^+^-ATPase providing its driving force, joint Na^+^,K^+^-ATPase/NKCC1 activity selectively imports K^+^ and Cl^−^ (and water). K^+^-stimulated NKCC1 activity will therefore help offset any intracellular K^+^ deficiency that might occur as a result of the asymmetry between the 3 Na^+^ molecules extruded and 2 K^+^ molecules accumulated for each molecule ATP converted to ADP, as will be discussed later. However, this may apply mainly or exclusively to astrocytes, since there is little evidence of functional importance of NKCC1 activation by high K^+^ concentration in neurons. Astrocytes express GABA_A_ receptors (Yoon et al., 2013), and since their chloride content is high, facilitation of GABA_A_ receptor stimulation by benzodiazepines leads to a depolarization, which might enable elevated opening of L-channels by lower than normal extracellular K^+^ concentrations (Hertz et al., 2006; Hertz and Chen, 2010). NKCC1 does appear also to be activated in many experimental conditions (perhaps often due to intense stimulation), where the NKCC1-mediated uptake of Na^+^,K^+^, 2 Cl^−^, and water leads to a short-lasting decrease of extracellular space, which is inhibitable by furosemide (Holthoff and Witte, 1996; Østby et al., 2009). Activation of the acid extruder Na^+^/bicarbonate cotransporter e1 (NBCe1) may contribute to the response (Østby et al., 2009), but a relatively small quantitative role of NBCe1 activation to the metabolic response to elevated extracellular K^+^ concentrations is discussed below. So is the importance of NKCC1 in regulatory volume increase during hypertonic conditions, which may be important during the undershoot of the extracellular K^+^ concentration. It will be shown below that in this situation Na^+^ accumulated by NKCC1 is not re-extruded, and the potential role of this phenomenon in counteracting the effects of the asymmetric ion transport by the Na^+^,K^+^-ATPase will be demonstrated in Table 2.

**Table 2:** **Gedankenexperiment considering effects of regulatory mechanisms on neuronal action potential-mediated neuronal Na^+^ uptake and K^+^ exit following uptake of 6 molecules of Na^+^ and release of 6 molecules of K^+^**.

**Location**	**Ion**	**Action potential- induced change**	**Astrocytic Na^+^, K^+^-ATPase**	**Na**_**x**_**-mediated Na^+^ uptake**	**Kir4.1-mediated K^+^release**	**Neuronal Na^+^, K^+^-ATPase**	**NKCC1-mediated uptake**	**Kir4.1-mediated K^+^ release**
Neuron	Na^+^	+6	+6	+6	+6	0	+1	+1
Neuron	K^+^	−6	−6	−6	−6	−2	−1	−1
Extracell	Na^+^	−6	+3	−3	−3	+3	0	0
Extracell	K^+^	+6	0	0	+6	+2	−1	0
Astrocyte	Na^+^	0	−9	−3	−3	−3	−1	−1
Astrocyte	K^+^	0	+6	+6	0	0	+2	+1

All values are shown as gain (+) or loss (−). The number of 6 was chosen as the least common denominator of 2 and 3, the numbers of K^+^ and Na^+^ ions, accumulated into, respectively released from cells during utilization of one molecule of ATP by the Na^+^,K^+^-ATPase. Alterations are shown in neurons, extracellular space and astrocytes after neuronal Na^+^ uptake, K^+^ release; K^+^-regulated activation of the astrocytic Na^+^,K^+^-ATPase; concomitant channel-mediated uptake of Na^+^, needed for stimulation of the intracellular Na^+^-sensitive site; numerically similar Kir4.1 K^+^ release (eliminating any charge change); Na^+^-regulated activation of the neuronal Na^+^,K^+^-ATPase; NKCC1- mediated Na^+^ and K^+^ uptake activated by, and eliminating extracellular hypertonicity and tentatively assumed to occur in astrocytes and neurons at a 2/1 ratio; and subsequent Kir4.1-mediated release from astrocytes of 1 K^+^, the number leading to correction of extracellular changes in Na^+^ and K^+^ concentrations and minimal intracellular changes. Evidence is found for the participation of all of these processes after action potential activity, but some of the values are arbitrary.

Increase in extracellular K^+^ concentration following intense GABAergic stimulation of neurons might be reversed by a similar mechanism as that described above, but whether this is the case is unknown. The increase occurs at a different region of the neurons and by a different mechanism, so the same clearance mechanisms are not necessarily involved. Moreover, intraneuronal Na^+^ is also increased and may cause sufficient stimulation of the neuronal Na^+^,K^+^-ATPase. The lack of certain demonstration of an increased extracellular K^+^ concentration after glutamatergic activation might indicate that no K^+^ is released extracellularly. However, in that case neuronal Na^+^,K^+^-ATPase activity should cause a decrease in extracellular K^+^ concentration, which has also not been described. Alternatively, channel-mediated release might occur but be compensated so rapidly by Na^+^,K^+^-ATPase-mediated neuronal re-uptake, greatly enhanced by the high intracellular Na^+^ concentration, that an extracellular increase becomes impossible to determine. It would be important to establish which of these two possibilities (if any), is correct since the mechanism by which intracellular Na^+^ concentration is normalized has implications for Na^+^,K^+^ balance and osmolarity both extra- and intracellularly.

### In summary

Cell culture experiments showed a faster active uptake of K^+^ in astrocytes than in neurons, a K^+^ stimulation of the astrocytic Na^+^,K^+^-ATPase (previously demonstrated in other astrocytic preparations) and involvement of Ca^2+^ in astrocytic K^+^ uptake. These findings led Mauro diNuzzo and coworkers to suggest that glycogenolysis is required for astrocytic K^+^ uptake. We confirmed this to be the case and demonstrated different glycogenolysis-dependent pathways for uptake of K^+^ concentrations ≤10 mM (where K^+^-stimulated activation of an ouabain pathway and glycogenolysis enabled Na^+^ entry for stimulation of the intracellular Na^+^-sensitive site of the Na^+^,K^+^-ATPase) and for NKCC1-mediated uptake at K^+^ concentrations above 10 mM. Accordingly astrocytic K^+^ uptake must stop after normalization of the extracellular K^+^ concentration, allowing K_ir_ 4.1-mediated K^+^ efflux, probably after trans-astrocytic K^+^ transport (Scemes and Spray, 2012), and reuptake into neurons, mediated by the less powerful, but not K^+^-stimulated neuronal Na^+^,K^+^-ATPase.

## Does extracellular hypertonicity elicit NKCC1-mediated ion/water uptake evoking volume increase and K^+^ undershoot?

**Figure 13:**
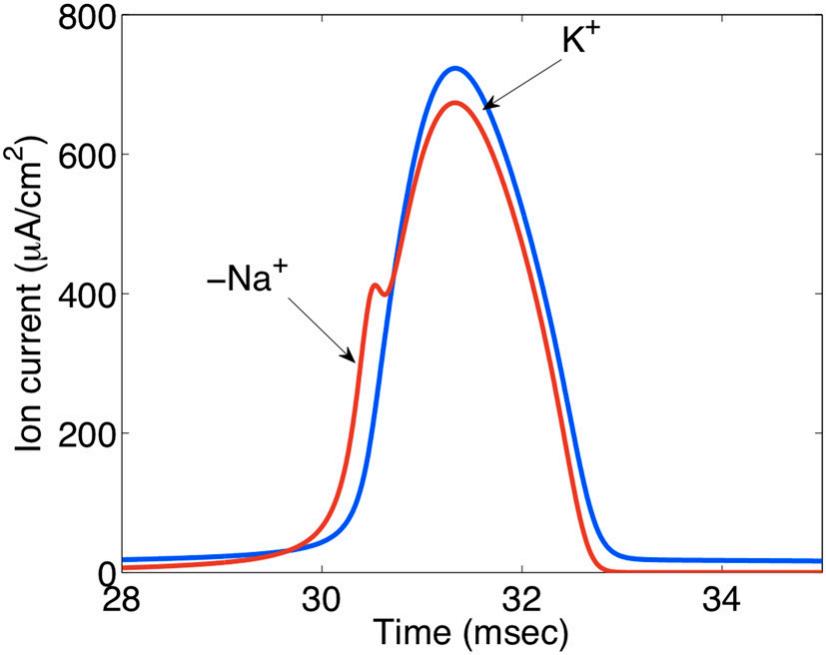
**Ion currents (μA/cm^2^) during action potential propagation in the squid giant axon.** The Na^+^ load per unit membrane area of an action potential in the squid axon is about 1168 nC/cm2 and the K^+^ load about 1343 nC/cm^2^. The negative Na^+^ is indicated by “−.” These relatively similar values contrast the 3/2 ratio between Na^+^ influx and K^+^ efflux during Na^+^,K^+^-ATPase-mediated active reversal of the events during action potential propagation. For this reason intense neuronal excitation followed by Na^+^,K^+^-mediated transport can be expected to lead to osmotic disturbances that will be most pronounced extracellularly because of the much smaller extracellular fluid space. Extracellular hyperosmolarity may, in turn, lead to cellular shrinkage that can be counteracted by NKCC1-mediated ion and water transport, creating additional energy expenditure and probably at least contributing to the post-excitatory undershoot. Gift from Moujahid.

**Figure 14:**
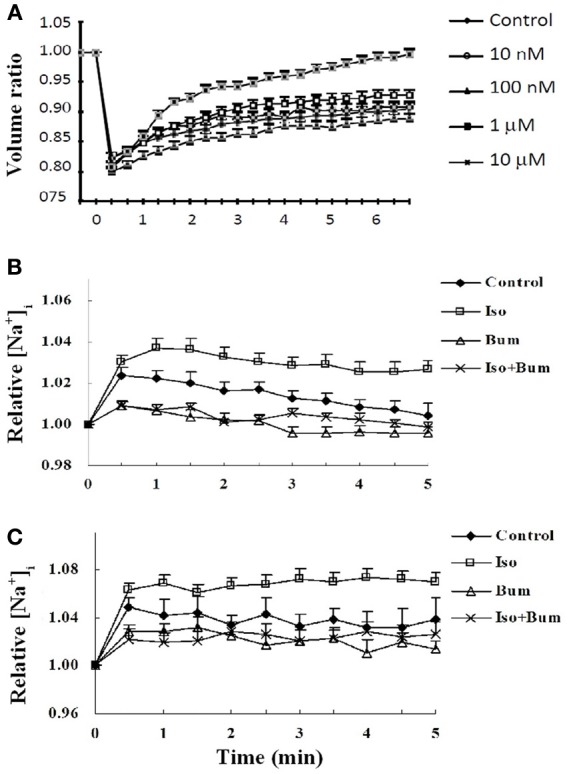
**Effect of isoproterenol on cell volume recovery and intracellular concentrations of K^+^ and Na^+^ in cultured astrocytes. (A)** After loading with calcein used as an indicator of intracellular water space, the cells were perfused in saline solution for 2 min. Thereafter, isotonic saline solution was changed to hyper-osmotic solution, obtained by adding sucrose to a final concentration of 100 mM, in the presence or absence of different concentrations of the β-adrenergic agonist isoproterenol. This can be seen to cause a ~20% decrease in cell volume, shown as a volume ratio between cell volume at the time of recording and cell volume before the hypertonic treatment, assigned a volume of 1. Under control conditions a slow regulatory volume increase occurs. This increase was concentration-dependently increased by isoproterenol, most marked at 10 μM. Additional experiments showed this to be a β1-adrenergic effect, inhibitable by furosemide, an inhibitor of the Na^+^,K^+^, 2 Cl^−^, and water cotransporter NKCC1. Results are averages from 42 to 77 cells on three-five separate coverslips. SEM values are indicated by vertical bars. **(B)** Effect of sucrose-induced hypertonicity on intracellular K^+^ concentrations in cultured astrocytes. The cells were treated for measurements of intracellular K^+^ concentration **(A)** as in Figure 11. Readings were made at 340 nm and 380 nm excitation and 510 nm emission at 15 s intervals, and the incubation continued for 5 min. Results are expressed in relation to control values, before addition of sucrose, assigned a value of 1. After addition of 100 mM sucrose at time zero the initial response of the K^+^ concentration to hypertonicity alone is about one half of that previously seen to an increase of extracellular K^+^ concentration by 5 mM (Xu et al., 2013). **(C)** Effect of sucrose-induced hypertonicity on intracellular Na^+^ concentrations in cultured astrocytes. The cells were treated for measurements of intracellular Na^+^ concentration with a different fluorescent drug, S-1264 SBFI-AM, and no addition of Pluronic F-127. The response of the Na^+^ concentration is almost identical and, if anything, slightly larger, indicating that Na^+^ accumulated by NKCC1 in this situation is not released by the Na^+^,K^+^-ATPase. For both K^+^ and Na^+^ the additional effect of isoproterenol (Iso) is relatively small and stable within the measuring period. Both with and without isoproterenol the increase in intracellular K^+^ or Na^+^ concentrations during cell volume recovery was virtually abolished by bumetanide (Bum). **(B)** and **(C)** show averages from 39 to 69 cells on two-four individual coverslips. SEM values are indicated by vertical bars.

A complete budget of metabolic costs of Na^+^,K^+^-ATPase-mediated K^+^ re-accumulation must also take the undershoot into account, although its dependence on intensive stimulation may suggest that the amount of ions transported is less than that during compensation for excitation-induced Na^+^ influx and K^+^ efflux (see also Table 2). The dependence of the undershoot on the intensity of previous excitation suggests that it could be caused either by the excitation as such or the subsequent normalization of the ionic imbalance by the activity of the Na^+^,K^+^-ATPase. Since Na^+^ influx and K^+^ efflux during the action potential are of similar magnitude (Figure 13) (Moujahid and d'Anjou, 2012; Moujahid, pers. commun.), whereas the Na^+^,K^+^-ATPase mediates Na^+^ efflux and K^+^ influx with a 3/2 ratio (Thomas, 1972; Clarke et al., 1989), osmotic imbalances may develop. These can be expected to be more pronounced extracellularly than intracellularly on account of the smaller extracellular fluid space, and Dietzel et al. (1982, 1989) showed extracellular hypertonicity after intense neuronal activity. Extracellular hypertonicity in brain is important, since Huang and Somjen (1995, 1997) demonstrated reduced neuronal excitability in brain slices exposed to hypertonic medium and also showed that the hypertonicity caused an increase in interstitial space of 18%, measured 30 min after addition of 25 mM mannitol. More recently Risher et al. (2009), have found an almost 30% decrease in astrocyte cell volume after 15 min of hyper-osmotic stress (+40 mOsm) in slices from transgenic mice expressing an astrocyte-specific marker. A regulatory volume increase in cultured astrocytes after addition of 100 mM sucrose and mediated by ion uptake (Pedersen et al., 2006) is slow in cultured astrocytes under control conditions, but greatly accelerated by the β-adrenergic drug isoproterenol (Figure 14A). Additional observations were (i) that activation of β1 receptors accounted for the response, and (ii) that it was abolished by the NKCC1 inhibitor bumetanide and by the Na^+^,K^+^-ATPase inhibitor ouabain. Figure 14B shows that the increase in volume after sucrose addition was achieved with only slight initial increases in Na^+^ and K^+^ concentrations, indicating equal uptake rates of Na^+^,K^+^, and water. It is peculiar that Na^+^,K^+^-ATPase activity providing the driving force for NKCC1 and stimulated by isoproterenol, in this situation does not extrude accumulated Na^+^. This may cast some doubt on the Na^+^ cycling theory between NKCC1 and the Na^+^,K^+^-ATPase, and a different possibility may be that K^+^-depolarization- and glycogenolysis-dependent opening of L-channels via complex Ca^2+^ signaling may activate Na^+^ uptake through transient receptor potential channels (TRPCs) as discussed by Yan et al. (2013) but only at highly elevated K^+^ concentrations. Further investigations showed dependence of regulatory volume increase on glycogenolysis, suggesting additional involvement of Na_x_ and Na^+^ uptake, similar to the situation when the Na^+^,K^+^-ATPase works independently of NKCC1. An additional increase in K^+^ and Na^+^ concentrations in the presence of isoproterenol was also small, and bumetanide caused almost complete inhibition, whether isoproterenol was present or not. The interpretation of these findings are that isoproterenol-mediated signaling stimulating the Na^+^,K^+^-ATPase required for NKCC1 function caused regulatory volume increase and in the process at the very least may contribute to the establishment of the undershoot in intact brain or brain slices (see Table 2 and later text). This conclusion is consistent with two previously discussed observations in intact brain tissue: inhibition of the undershoot by furosemide (Figure 4), and dependence of the undershoot on intact IP_3_ receptor function (Figure 7), a receptor operating within the β_1_-adrenergic signaling pathway in astrocytes (Du et al., 2010).

It cannot be excluded that α_2_-adrenergic stimulation of the neuronal Na^+^,K^+^-ATPase also contributes to the undershoot, since another co-transporter, KCC2 which is expressed in neurons, is also blocked by furosemide (Russell, 2000; Blaesse et al., 2009). However, at least under the conditions used by Viitanen et al. (2010) this co-transporter released K^+^, which would increase, not decrease the undershoot. More importantly, the demonstration of NKCC1 expression in neuronal dendrites (Marty et al., 2002) may allow neurons to participate in transmitter-regulated volume increase, since their Na^+^,K^+^-ATPase is activated by α_2_-adrenergic stimulation (Hajek et al., 1996). Table 2 shows a Gedankenexperiment (thought experiment) evaluating the cationic consequences of the additional action of NKCC1 after action-potential-mediated entry/exit of 6 (the least common denominator of 2 and 3) Na^+^ and K^+^, subsequent astrocytic K^+^-activated Na^+^,K^+^-ATPase activity, dependent upon channel-mediated Na^+^ influx, K_ir_ 4.1-mediated K^+^ release, neuronal Na^+^-activated Na^+^,K^+^-ATPase activity. Assuming that the activity of NKCC1 leads to astrocytic uptake of 2 K^+^ and 2 Na^+^ and neuronal uptake of 1 K^+^ and 1 Na^+^, and that one astrocytic K^+^ is subsequently released through K_ir_4.1 channels [as indicated by Ba^2+^ sensitivity of the undershoot shown *in vivo* (Figure 2)] the end result will be remarkably small ionic deviations in neurons, astrocytes and extracellular fluid. Other processes, including glutamate/ion cotransport may further modify the ionic alterations. Moreover, Table 2 does not include ion fluxes activated by glutamatergic neuronal excitation.

### In summary

The asymmetry between Na^+^,K^+^-ATPase stimulated Na^+^ efflux and K^+^ influx creates extracellular hypertonicity, leading to pronounced astrocytic shrinkage. Transmitter-mediated stimulation of the Na^+^K^+^-ATPase/NKCC1 transporter promotes regulatory volume increase, at the very least contributing to the post-excitatory K^+^ undershoot and helping to correct ionic disequilibria caused by the asymmetrical Na^+^ and K^+^ fluxes.

## Comparison between effects on energy metabolism by K^+^/Na^+^ transport compared to other K^+^-mediated effects

**Figure 15:**
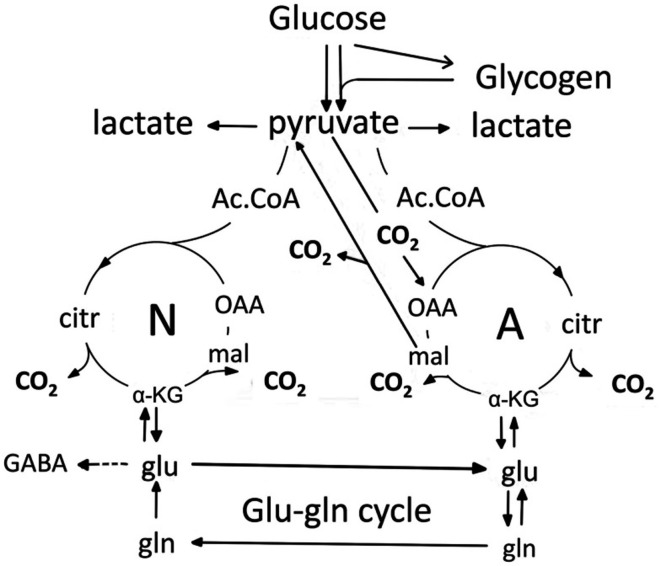
**Cartoon of glucose metabolism via pyruvate in neurons (left—N) and astrocytes (right—A) and of glutamine-glutamate(GABA) cycling.** Glycolysis, i.e., formation of pyruvate from glucose produces a small amount of energy (2 ATP for each molecule of glucose), and there is no firm evidence glycolytically derived energy is needed for ion transport in either neurons or astrocytes. However, pyruvate formation can also occur via glycogen, but only in astrocytes. Its degradation, glycogenolysis, is extremely important for K^+^ uptake in astrocytes because K^+^-stimulated glycogenolysis is essential for the initial clearance of elevated extracellular concentrations by the astrocytic Na^+^,K^+^-ATPase and transmitter-mediated glycogenolysis is needed for the Na^+^,K^+^-ATPase/NKCC1-mediated undershoot. In both cell types pyruvate metabolism via acetyl Coenzyme A (ac.CoA) leads to formation of citrate by condensation with pre-existing oxaloacetate (OAA) in the tricarboxylic acid (TCA), an end-result of the previous turn of the cycle. Citrate oxidation in the TCA cycle includes two decarboxylations, leading to reformation of oxaloacetate, ready for another turn of the cycle, and to production of large amounts of energy (~30 ATP). Pyruvate carboxylation creates a new molecule of oxaloacetate, which after condensation with acetyl Coenzyme A, derived from a second molecule of pyruvate, forms a new molecule of citrate. This process can be used for replacement of worn TCA cycle intermediates. More important for brain function is that α-ketoglutarate (α-KG), one of the intermediates of the TCA cycle can leave the cycle to form glutamate (glu) and, catalyzed by the cytosolic and astrocyte-specific enzyme glutamine synthetase, glutamine (gln). After release from astrocytes glutamine is accumulated in glutamatergic and GABAergic neurons [lower line of the glutamineglutamate (GABA) cycle] (glu-gln cycle), converted to glutamate (and in GABAergic cells onwards to GABA) and released as transmitter. Released glutamate is almost quantitatively re-accumulated in astrocytes, together with part of the released GABA and reaccumulated in the astrocytic cytosol. Here, about 85% is converted to glutamine and reenters the glutamine-glutamate(GABA) cycle. Although the figure may give the impression that this process is not influenced by astrocytes, this may be incorrect, since glutamine may undergo trans-astrocytic transport. The remaining 15% is oxidatively degraded after re-conversion via α-ketoglutarate to malate, exit of malate to the cytosol, decarboxykation to pyruvate by the cytosolic malic enzyme and further pyruvate oxidation in the TCA cycle via acetyl Coenzyme A. Combined astrocytic formation and oxidation of glutamate creates almost as much ATP as direct oxidation of glutamate (Hertz et al., 2007).

All active transport mechanisms functioning during normalization of cellular and extracellular ion distribution after action potential-mediated neuronal Na^+^ entry and K^+^ exit must obviously be taken into account when evaluating metabolic effects of action potential propagation. The “budgets” to be discussed later clearly identify ion transport, either secondary to glutamatergic stimulation or caused by action potential propagation as the metabolic costliest processes in brain. This section will describe K^+^ effects on astrocytic and neuronal glucose metabolism. Relevant aspects of glucose metabolism in neurons and astrocytes are illustrated in Figure 15 and described in more detail in its legend. Highly elevated K^+^ concentrations (>15–20 mM) have repeatedly been found to cause large, but transient increases in rate of oxygen consumption in brain slices (Ashford and Dixon, 1935; Dickens and Greville, 1935; Hertz and Schou, 1962), freshly dissected glial cells (Hertz, 1966; Aleksidze and Blomstrand, 1969) or cultured astrocytes (Hertz et al., 1973). The K^+^ concentrations used, and shown to be necessary for the response in brain slices, have generally been those now known to stimulate NKCC1 (Hertz et al., 2004). The brain slice response to elevated K^+^ concentrations is exerted on astrocytes (Badar-Goffer et al., 1992). Electrical stimulation causes similar increases in brain slice respiration, probably acting mainly or exclusively on neurons. The reason lower K^+^ concentrations, selectively stimulating the Na^+^,K^+^-ATPase, cause little or no stimulation of oxygen consumption in brain slices and cultured astrocytes [isolated and incubated preparations with easy lactate exit Ashford and Dixon, 1935; Walz and Mukerji, 1988] may be that glycolysis is greatly enhanced due to a large lactate efflux to the huge incubation medium. As a result feed back inhibition of glycolysis may be inhibited and a small stimulation of the resulting high rate of glycolysis may provide sufficient energy for K^+^ uptake, when the rise in K^+^ concentrations is modest. Moreover, at low increases in extracellular K^+^ the increase in energy metabolism is evoked exclusively by increased ADP (as a result of increased energy demand). When energy metabolism is further stimulated, and perhaps especially when cytosolic and mitochondrial Ca^2+^ entry at extracellular K^+^ concentrations ≥10 mM is increased, leading to direct activation of mitochondrial enzymes (Denton, 2009) the increase in oxidative metabolism becomes obvious. K^+^ concentrations of 10–12 mM stimulate glycolysis in cultured astrocytes, with >80% of the stimulation evoked by a rise in extracellular K^+^ concentration from 5.4 to 12 mM being inhibited in the presence of 10 mM ouabain (Peng et al., 1996). Higher K^+^ concentrations increase oxidative glucose metabolism in cultured astrocytes (Hertz et al., 1973).

The preferential (Roberts, 1993) or selective (Peng et al., 1996) use of glycolysis for ion transport observed in brain slices or cultured astrocytes does not mean that either neurons (Chih et al., 2001) or astrocytes require glycolysis for uptake of K^+^ in the range 5–10 mM. Thus, K^+^ uptake proceeds equally well in cultures where inhibition of glycolysis by 2-deoxyglucose (which abolishes K^+^ uptake) is compensated for by addition of pyruvate. In intact brain utilization of labeled acetate, an astrocyte-specific mitochondrial substrate is increased during brain activation, e.g., by photic, acoustic, or electrical stimulation (Cruz et al., 2005; Dienel et al., 2007; Wyss et al., 2009, 2011). During spreading depression, with large increase in extracellular K^+^ concentration (stimulating NKCC1), this response is greatly enhanced (Dienel et al., 2001).

A report by Bittner et al. (2011) of huge K^+^-induced increases in glycolysis in cultured astrocytes is disputable, due to very low glucose analog concentration used during the experiments and changes in virtually all concentrations (e.g., glucose, K^+^) between culturing and control conditions (Porras et al., 2004; Bittner et al., 2010). Such a procedure severely violates the principle of an intact milieu interne. Higher rates of glucose uptake in astrocytes than in neurons were also determined in brain slices in a collaboration between the same group (Barros et al., 2009; Barros, 2013; Jakoby et al., 2013) and that of Deitmer. On one hand this could be due to the use of very young animals, where metabolism is very different from that in mature animals [reviewed by Hertz et al. (2013)]. On the other hand, it might also be related to an overall higher expression of most enzymes involved in glucose metabolism in glial cells (Lovatt et al., 2007) and to the high rate of glycolysis and glucose release (see above) in brain slices.

A postulate by Ruminot et al. (2011) from the Barros group that stimulation of NBCe1 in response to an increase in intracellular pH is the only reason for the increase of astrocytic glycolysis during exposure to an elevated concentration of extracellular K^+^ is also suspect, although the enhanced acid extruder activity does contribute to the response. Brookes and Turner (1994) found that an increase in the extracellular K^+^ concentration from 3 to 12 mM increased pH_i_ in cultured astrocytes by 0.28 pH units by depolarization-mediated stimulation of influx of a Na^+^/bicarbonate cotransporter, importing at least two HCO^−^_3_ with each Na^+^. We have confirmed these results, demonstrated mRNA expression of NBCe1, a Na^+^/bicarbonate cotransporter with these characteristics, in our cells, and measured an increase in pH_i_ of 0.13 when the extracellular K^+^ concentration was increased from 3 to 5.4 mM and of 0.29 when it was increased to 12 mM We have also determined that the so-called molar extrusion rate of H^+^ (J_*H*+_) mediated by NBCe1 (in reality bicarbonate influx rate) increases from ~40 μM/sec under control conditions (5.4 mM K^+^) to ~3 times this value (~120 μM/sec), when gradual drug-induced up-regulation of the NBCe1 gene increases ΔpH_i_/Δt to a rate (0.62) which greatly exceeds that (0.28 or 0.29), occurring when the extracellular K^+^ concentration is raised from 5 to 12 mM. This increase in J_*H*+_ is almost comparable to that which can be calculated from the K^+^-induced increase in K^+^ influx in Figure 8 during a similar change in [K^+^]_o_, based on a fluid/protein ratio in these cultures of ~4 (Chen et al., 1992). However, due to the different energy demand by the two transporters. Energy use is much less for pH regulation, although—and then continue as is with brain energy metabolism is also increased by the increase in pH as such (mainly due to an increase in phosphofructokinase activity), but this increase can be expected to be small compared to that evoked by the increased pumping of Na^+^ and K^+^ caused by the increase in extracellular K^+^ concentration. This conclusion is based upon relatively small increases in brain slice oxygen consumption by an increase in pH of ≤0.3 above control conditions (Canzanelli et al., 1939; Elliott and Birmingham, 1949). Thus the conclusion stands that the metabolic stimulation by a moderate increase of the extracellular K^+^ concentration is mainly, although not exclusively, due to stimulation of active transport of K^+^ (Song et al., under revision).

Extracellular K^+^ concentrations of 10–12 mM are not high enough to sufficiently depolarize cultured neurons. Higher K^+^ concentrations (25 and 50 mM) induce glutamate release in cultured glutamatergic neurons (cerebellar granule cells) (Peng et al., 1991), which is associated with a large increase (doubling) in rate of oxidative metabolism, an increase which is almost completely inhibited by ouabain and mimicked by monensin (Peng, 1995; Peng et al., 1996). That 12 mM extracellular K^+^ selectively stimulates astrocytic metabolism and higher K^+^ concentrations are required to stimulate neuronal metabolism is consistent with findings in neuronal and mixed neuronal glial cultures by Honegger and Pardo (1999). Thus, during stimulation a Na^+^ extrusion rate of >15 mM/min under control conditions (corresponding to a K^+^ uptake rate of >10 mM/min) might increase to >30 mM/min.

Elevated K^+^ concentrations also increase pyruvate carboxylation (Kaufman and Driscoll, 1992), but the effect on energy metabolism is probably minor, since total glutamate turnover was associated with relatively little demand for energy.

### In summary

By far the major metabolic effect of elevated K^+^ concentrations on astrocytes is a reflection of the increased active uptake of K^+^. Minor increases in extracellular K^+^ concentration do not stimulate neuronal metabolism.

## Correlation with known contribution of astrocytes to brain oxidative metabolism *in vivo* and with Attwell's budgets

Beginning with pioneering studies by Gruetter et al. (2001) and Lebon et al. (2002) a series of investigations using ^13^C- or in some cases ^11^C-nuclear magnetic resonance (NMR) imaging has very consistently found that oxidative metabolism in glial cells (in all probability exclusively astrocytes) in the human and rodent brain cortex *in vivo* accounts for 20–25% of total oxidative metabolism (reviewed and tabulated by Hertz, 2011). However, in the Attwell budgets (Attwell and Laughlin, 2001; Howarth et al., 2012) the only glial contribution is part of the 4% attributed to turnover of glutamate via the glutamate-glutamine cycle. The NMR studies in addition showed that glutamate-glutamine cycle fluxes almost equal rates of glucose metabolism in brain. Calculations using these rates yield results for energy expenditure by glutamate-glutamine cycling that are reasonably consistent with the 4% of total energy consumption calculated in the Attwell budgets (perhaps slightly higher). This low percentage is an indication of the enormity of the other energy-consuming processes going on in brain, first and foremost transport of Na^+^ and K^+^.

Because the temporal overlap between Na^+^ and K^+^ currents during an action potential was recently found by Alle et al. (2009) and Carter and Bean (2009) (see also Sengupta et al., 2010) to be much less than originally estimated, the relative contribution by action potentials was re-calculated in the most recent Attwell budget (Howarth et al., 2012) and reduced to 16%. This left synaptic transmission, i.e., mainly glutamatergic signaling, as the costliest energetic process occurring in brain, and a total energy consumption for maintenance of resting potential, action potential and synaptic transmission of 20.4 μmol ATP/g per min. This total calculated energy consumption was compared to a total rate of energy use in the gray matter of rat of 33–50 μmol ATP/g per min, based on data by Sokoloff et al. (1977). Especially the higher of these rates, representing the cortical structures with the highest rate of glucose utilization, considerably exceed a possibly more realistic rate of 22.4 μmol/g per min, based on an average resting cortical glucose utilization of 0.7 μmol/g per min (Rothman et al., 2011) and an ATP yield of 30 (oxidatively derived) +2 (glycolytically derived) ATP—not 36 +2 as previously assumed—generated by oxidation of one molecule of glucose. This would leave space for householding expenses (e.g., protein synthesis) of 10%, which appears reasonable, considering the estimate by Rothman et al. (2011) that signaling processes account for at least 80% of energy metabolism in brain cortex. The budget would leave no room for the metabolic costs of an additional astrocytic contribution to K^+^ uptake and for subsequent Na^+^,K^+^-ATPase-driven NKCC1 operation. However, the very high estimate for synaptic transmission might be an overestimate, since the literature data on which Attwell and Laughlin (2001) based their estimate of the energetic cost of synaptic transmission, were quite variable. Nevertheless, that synaptic transmission contributes much to total energy metabolism is consistent with a larger contribution of dendrites to total energy metabolism in brain than to total volume [reviewed by Hertz (2011)], since dendritic Na^+^ concentration increases greatly as a result of synaptic stimulation (Lasser-Ross and Ross, 1992; Rose and Konnerth, 2001; Bennay et al., 2008). However, in spite of Na^+^,K^+^-ATPase-mediated reversal of the large neuronal Na^+^ uptake being the main reason for the high energetic costs of glutamatergic synaptic transmission it may not trigger an initial astrocytic uptake. As already mentioned, the neuronal Na^+^,K^+^-ATPase may be sufficiently stimulated by the increased intracellular Na^+^ concentration for an efficient neuronal Na^+^ re-extrusion to occur, combined with a fast K^+^ uptake. The experiments by Ransom et al. (2000) dealt exclusively with action potentials, and up to 16% of total energy consumption for astrocytic uptake of K^+^ during action potentials plus a few percent for astrocytic contribution to glutamate recycling and another astrocytic energy consumption during the undershoot would together account for the ~25% of total brain energy metabolism, known to be astrocytic [see review by Hertz (2011)]. This conclusion is consistent with the lack of data showing increase in extracellular K^+^ concentration in response to glutamatergic stimulation of synaptic transmission.

The increase in extracellular K^+^ concentration during intense GABAergic neuronal stimulation may cause some stimulation also of the astrocytic Na^+^,K^+^-ATPase, but it should be remembered that in this case intraneuronal Na^+^ concentration is also elevated.

### In summary

The Attwell budgets probably greatly underestimate astrocytic contributions. The underestimates may be major in the case of action potential propagation, but of unknown magnitude for the metabolic response to glutamatergic stimulation.

## Glutamate/glutamine/glutamate cycling provides precedence for sequential cellular uptakes

K^+^ is not alone in probably being accumulated first by astrocytes, released and re-accumulated into neurons, a metabolically costly procedure which must provide advantages for brain function in order to be justified. It is generally acknowledged that after its release from neurons glutamate is almost exclusively accumulated in astrocytes (Danbolt, 2001). After conversion to glutamine, which may traverse part of the astrocytic network (Cruz et al., 2007) glutamine is released from astrocytes and taken up in neurons. Two different types of transporters are used for release from astrocytes and uptake in neurons, securing a cellularly directed transport (Boulland et al., 2002; Jenstad et al., 2009). The whole course constitutes a glutamate-glutamine shuttle, which includes several processes requiring energy-expenditure, first active uptake of glutamate in astrocytes (Danbolt, 2001) and its subsequent amidation to glutamine (Norenberg and Martinez-Hernandez, 1979), next uptake of glutamine in neurons, followed by its deamidation to glutamate (Palaiologos et al., 1989; Bak et al., 2008), and eventually accumulation into synaptic vesicles by a vesicular transporter (Maycox et al., 1988; Herzog et al., 2006; Sreedharan et al., 2010). Moreover, as described in more detail in the legend of Figure 15 neurons are unable to metabolize glucose to glutamate, so that glutamate must initially be formed in astrocytes (Yu et al., 1983; Shank et al., 1985), requiring Ca^2+^, K^+^ and transmitter dependent pyruvate carboxylation (Garrison and Borland, 1979; Kaufman and Driscoll, 1992). Disregarding the huge importance of glutamatergic excitation on neuronal metabolism, the whole process operating during synthesis, cellular transfer and metabolism of transmitter glutamate (and GABA) has been estimated to account for only about 4% of total energy metabolism in cerebral cortex (Howarth et al., 2012). This reflects that glutamate as a transmitter is released in relatively small amounts, compared to Na^+^ and K^+^ turnover during neuronal excitation. However, the amount of glutamate traveling in the glutamate-glutamine shuttle is large and almost equals the total amount of oxidative glucose utilization (Sibson et al., 1998; Rothman et al., 2011; Hertz, 2013). The relatively low contribution to total energy requirement by glutamate turnover is therefore an indication of the colossal amount of Na^+^ and K^+^ pumping in the brain. It is thought-provoking that both K^+^ and glutamate seem to utilize double metabolic “billing” for their uptakes. However, although glutamate's own metabolism requires so relatively little energy, its possible trans-astrocytic transport may exert a huge effect on local energy metabolism, because of the pronounced influence of glutamatergic signaling on ion homeostasis. Again it would be of paramount importance to track potential astrocytic trafficking by computational methods.

### In summary

Glutamatergic signaling is well known to involve both astrocytic and neuronal processes and sequential, energy-consuming uptakes in both cell types. It may be of special importance that glutamine, like K^+^ can travel between connexin- and/or pannexin-coupled astrocytes.

## Can astrocytic/neuronal sequential Na^+^/K^+^ pumping and NKCC1-mediated regulatory volume increase yield functional gains?

A recent paper (Hertz, 2013) discussed potential functional advantages gained by the brain in return for the metabolic “double-dipping” caused by the costly glutamate-glutamine cycling. Glutamine transfer between gap-junction coupled astrocytes was pointed out as a potential important factor, and postnatal development of the neuronal-astrocytic system was emphasized. It has long been known that both K^+^ and Na^+^ traverse the functional astrocytic syncytium via gap junctions (McKhann et al., 1997; Rose and Ransom, 1997). Enhanced activity of both connexin- and pannexin-mediated channels by moderate elevations in extracellular K^+^ is likely to have profound effects on intercellular signaling among astrocytes (Scemes and Spray, 2012). In hippocampal slices from mature mice the immediate increase in intracellular Na^+^ concentration in a single astrocyte exposed to direct electrical stimulation thus spreads by diffusion to neighboring astrocytes within a distance of 100 μm (Langer et al., 2012). Brain network models that are not anatomically based (e.g., Moujahid et al., 2011) could be instrumental in revealing functional importance of trans-astrocytic transport. Channel-mediated Na^+^ redistribution was reduced in slices from 4-day-old mice (Rose and Ransom, 1997), reflecting paucity and immaturity of astrocytes at early postnatal ages, where several enzymes also show different cellular expression [reviewed by Hertz (2013)]. K^+^ uptake in slices from 2 week-old rats is also not inhibited by 2 mM Ba^2+^ (Gabriel et al., 1998).

It was mentioned above that extracellular hypertonicity decreases neuronal excitability (Huang and Somjen, 1995, 1997). No information is available about the mechanism(s) involved, but studies by Yuan et al. (2010) have demonstrated that neuronal expression of Fos protein *in vivo* in response to acute hypertonicity depends on previous activation, and preceding Fos expression in astrocytes and are blocked by the primarily astrocytic toxin fluoroacetate. We have also discussed that hypertonicity induces cell shrinkage, and that regulatory volume increase of astrocytes mediated by the Na^+^,K^+^-ATPase/NKCC1 ion and water transport system and accelerated by β-adrenergic stimulation at least contributed to the undershoot. Slow neuronal hyperpolarization (sAHP) has effects that strikingly remind of those evoked by hypertonicity, and McCormick and Prince (1988) described that β-adrenergic termination of sAHP in isolated thalamic tissue was abolished at an elevated extracellular K^+^ concentration. These characteristics are similar to those of β-adrenergic stimulation of the astrocytic, but not the neuronal Na^+^,K^+^-ATPase, since the latter is not activated by β-adrenergic, but by α_2_-adrenergic activation. It is therefore possible that β-adrenergic acceleration of regulatory volume increase in astrocytes (Figure 14) may be important for termination of sAHP. If this is the case, behavioral effects, including those involved in learning (Oh et al., 2009), that depend upon neuronal-astrocytic interactions during restoration of normal ion distribution after action potential-mediated increases in extracellular K^+^ concentration would be immense. The studies by McCormick and Prince (1988) point in that direction, and astrocytic involvement is supported by the following presently reviewed findings. (i) Although increase of extracellular K^+^ concentration abolishes noradrenaline stimulation of the Na^+^,K^+^-ATPase in both neurons and astrocytes, the β-adrenergic effect is astrocyte-specific. (ii) Activation of the Na^+^,K^+^-ATPase/NKCC1 ion/water transport system by isoproterenol greatly enhances astrocytic volume recovery during hypertonic shrinkage. (iii) Exposure to hypertonicity evokes neuronal effects in intact tissue corresponding to those of hypertonicity. (iv) A transmitter effect on astrocytes can reduce extracellular K^+^ concentration in intact tissue. And (v) NKCC1-mediated, probably mainly astrocytic ion transport contributes to the undershoot in intact tissue and thus occurs simultaneously with sAHP and its termination.

### In summary

Trans-astrocytic transport may provide signaling possibilities. Potential acceleration of the termination of slow neuronal hyperpolarization by transmitter-mediated stimulation of the Na^+^,K^+^-ATPase/NKCC1 transporter would have wide-ranging behavioral consequences. Literature data obtained in intact tissue are in accordance with such an effect.

## Concluding remarks

Increasing evidence supports the concept that cellular K^+^ re-accumulation following increases in extracellular K^+^ concentrations during excitatory activity in the adult mammalian brain occurs by an initial uptake into astrocytes. This seems to be followed by channel-mediated K^+^ release to the extracellular space, probably after trans-astrocytic transport, and subsequent re-accumulation by neurons and by eventual NKCC1-mediated regulatory volume increase (Table 2). Together these processes may almost correct ionic disequilibrium resulting from equal fluxes of Na^+^ and K^+^ during action potential propagation but not during its reversal by the Na^+^,K^+^-ATPase. Attempts to quantify the metabolic cost associated of signaling activity must take this possibility into account. Network models that are not based on anatomical connections may be crucial for determination of the importance of the suggested trans-astrocytic transport. Trans-astrocytic K^+^ transport may provide signaling opportunities and regulation of sAHP means of regulation of neuronal excitability, which could justify the increased metabolic cost of sequential astrocytic and neuronal K^+^ uptake.

### Conflict of interest statement

The authors declare that the research was conducted in the absence of any commercial or financial relationships that could be construed as a potential conflict of interest.
